# Unlocking secrets of microbial ecotoxicology: recent achievements and future challenges

**DOI:** 10.1093/femsec/fiad102

**Published:** 2023-09-05

**Authors:** Jennifer Hellal, Lise Barthelmebs, Annette Bérard, Aurélie Cébron, Giulia Cheloni, Simon Colas, Cristiana Cravo-Laureau, Caroline De Clerck, Nicolas Gallois, Marina Hery, Fabrice Martin-Laurent, Jean Martins, Soizic Morin, Carmen Palacios, Stéphane Pesce, Agnès Richaume, Stéphane Vuilleumier

**Affiliations:** BRGM, F-45071 Orléans, France; Université de Perpignan Via Domitia, Biocapteurs – Analyse-Environnement, Perpignan, France; Laboratoire de Biodiversité et Biotechnologies Microbiennes, USR 3579 Sorbonne Universités (UPMC) Paris 6 et CNRS Observatoire Océanologique, Banyuls-sur-Mer, France; UMR EMMAH INRAE/AU – équipe SWIFT, 228, route de l'Aérodrome, 84914 Avignon Cedex 9, France; Université de Lorraine, CNRS, LIEC, 54000 Nancy, France; MARBEC, Univ Montpellier, CNRS, Ifremer, IRD, Sète, France; Universite de Pau et des Pays de l'Adour, E2S UPPA, CNRS, IPREM, Pau, France; Universite de Pau et des Pays de l'Adour, E2S UPPA, CNRS, IPREM, Pau, France; AgricultureIsLife, Gembloux Agro-Bio Tech (Liege University), Passage des Déportés 2, 5030 Gembloux, Belgium; Université de Lorraine, CNRS, LIEC, 54000 Nancy, France; HydroSciences Montpellier, Université de Montpellier, CNRS, IRD, Montpellier, France; Institut Agro Dijon, INRAE, Université de Bourgogne, Université de Bourgogne Franche-Comté, Agroécologie, 21065 Dijon, France; IGE, UMR 5001, Université Grenoble Alpes, CNRS, G-INP, INRAE, IRD Grenoble, France; INRAE, UR EABX, F33612 Cestas cedex, France; Université de Perpignan Via Domitia, CEFREM, F-66860 Perpignan, France; CNRS, CEFREM, UMR5110, F-66860 Perpignan, France; INRAE, UR RiverLy, Villeurbanne, France; Université de Lyon, Université Claude Bernard Lyon 1, CNRS, UMR 5557, Ecologie Microbienne, Villeurbanne, France; GMGM, UMR 7156 Université de Strasbourg – CNRS, Strasbourg, France

**Keywords:** diversity, ecosystem functions, holobiont, microorganisms, nature-based solutions, pollution

## Abstract

Environmental pollution is one of the main challenges faced by humanity. By their ubiquity and vast range of metabolic capabilities, microorganisms are affected by pollution with consequences on their host organisms and on the functioning of their environment. They also play key roles in the fate of pollutants through the degradation, transformation, and transfer of organic or inorganic compounds. Thus, they are crucial for the development of nature-based solutions to reduce pollution and of bio-based solutions for environmental risk assessment of chemicals. At the intersection between microbial ecology, toxicology, and biogeochemistry, microbial ecotoxicology is a fast-expanding research area aiming to decipher the interactions between pollutants and microorganisms. This perspective paper gives an overview of the main research challenges identified by the Ecotoxicomic network within the emerging One Health framework and in the light of ongoing interest in biological approaches to environmental remediation and of the current state of the art in microbial ecology. We highlight prevailing knowledge gaps and pitfalls in exploring complex interactions among microorganisms and their environment in the context of chemical pollution and pinpoint areas of research where future efforts are needed.

## Teaser

Overview of the main research challenges at different scales for microbial ecotoxicology as identified by the Ecotoxicomic network in the light of the current state of the art.

## Introduction

**Figure 1.**
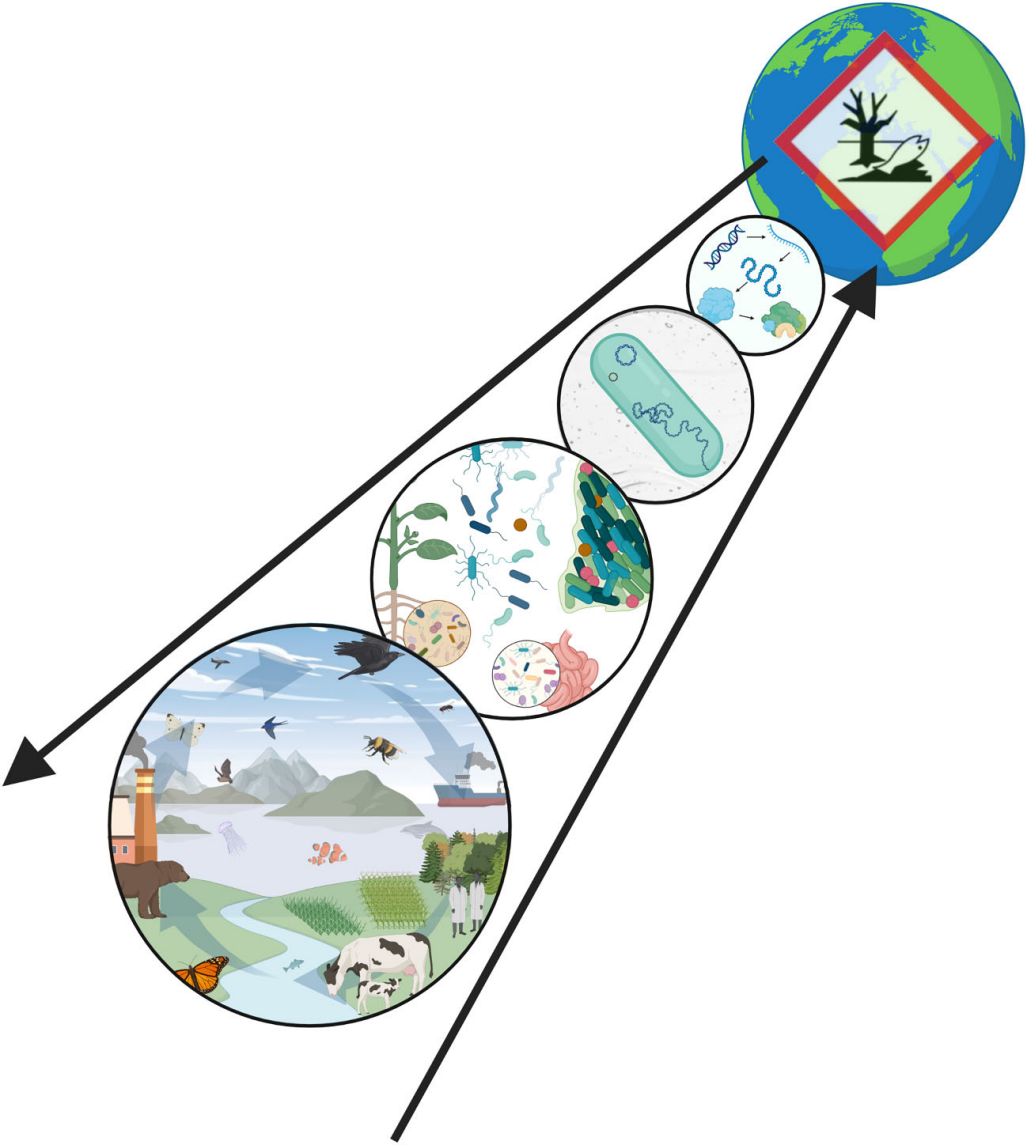
The multiple scales of microbial ecotoxicology, at molecular, cellular, community (including interactions), and ecosystem levels.

Ecotoxicology is defined as the "study of the toxic effects of chemical and physical agents on all living organisms, especially on populations and communities within defined ecosystems; it includes transfer pathways of these agents and their interactions with the environment", whereas ecology is defined as the "branch of biology that studies the interactions between living organisms and all factors (including other organisms) in their environment. Such interactions encompass environmental factors that determine the distributions of living organisms" (Nordberg et al. 2009). In the Anthropocene, environmental pollution is omnipresent alongside other environmental factors. In order to understand the impacts of chemical pollution and their consequences on the interactions between organisms and their environment, ecotoxicology relies on existing ecological theories whereas in ecology, pollution is only one factor amongst many others. In this sense, ecotoxicology rather than ecology is relevant for environmental regulatory issues and for environmental risk assessment (ERA). In microbial ecology, this has led to the emergence of a fast-expanding research area, microbial ecotoxicology, at the intersection between microbial ecology, toxicology, and biogeochemistry that aims to decipher the interactions between pollutants and microorganisms at different organizational scales (Ghiglione et al. 2014, 2016). Interdisciplinarity is, thus both a key feature and a requirement in microbial ecotoxicology studies and for applications of newly generated knowledge for toxicity assessment and environmental remediation. In this context, microbial ecotoxicology builds on a paradox in several ways (Fig. 1). First, it strives to yield insights about pollutant-driven impacts on ecosystem functioning at the global scale based on micrometer-scale processes. Second, in order to do so, it strongly relies on existing knowledge and detailed analysis of individual model microorganisms to characterize the response of complex microbial communities. Moreover, it is developing an increasing interest in testing and applying concepts developed for classical macroecology, e.g. functional traits, tolerance, resistance, functional redundancy, resilience, and connectivity (Cébron et al. 2021, Romillac and Santorufo 2021, Mony et al. 2022) through investigations to comprehend higher levels of organization at the community and ecosystem scale (Loreau et al. 2001). A better understanding of the complex interactions between microbial communities and pollutants is essential for toxicity assessment and the implementation of sustainable bioremediation systems, i.e. the use of nature-based solutions to eliminate pollution. Of course, several scientific challenges are still being actively tackled to enable a wider use of microorganisms in these fields (Peixoto et al. 2022). This also includes the development and application of new technologies and methods in microbial ecology to isolate and functionally characterize a larger diversity of microorganisms from environmental samples (Duran et al. 2022).

To meet these ambitious expectations, microbial ecotoxicology will benefit from the EcotoxicoMic network (https://ecotoxicomic.org/) born in France as a national network in 2013, which has now reached an international dimension (Pesce et al. 2020a, Gallois et al. 2022). This perspective paper aims to present the main challenges and research opportunities identified for microbial ecotoxicology in light of the current state of the art. We focused on organic and inorganic chemical pollutants, leaving aside topics associated with pathogens, antibiotics, and microbially produced toxins that would warrant a specific discussion. The first two sections present the heart of microbial ecotoxicology and consider the impacts of pollutants on microbial biodiversity and functions and then the role of microorganisms in pollutant transformation, biodegradation, and transfer. The third section addresses the major challenge of linking the impact of pollution on microorganisms with the functioning of hosts and ecosystems and possible consequences at a global scale. Finally, the fourth section provides an overview of current applications of microbial ecotoxicology for practical environmental assessment and bioremediation and associated challenges. Methods and technologies applied or considered in the field of microbial ecotoxicology today are discussed throughout the paper.

## Impacts of pollutants on microbial biodiversity and functions

**Figure 2.**
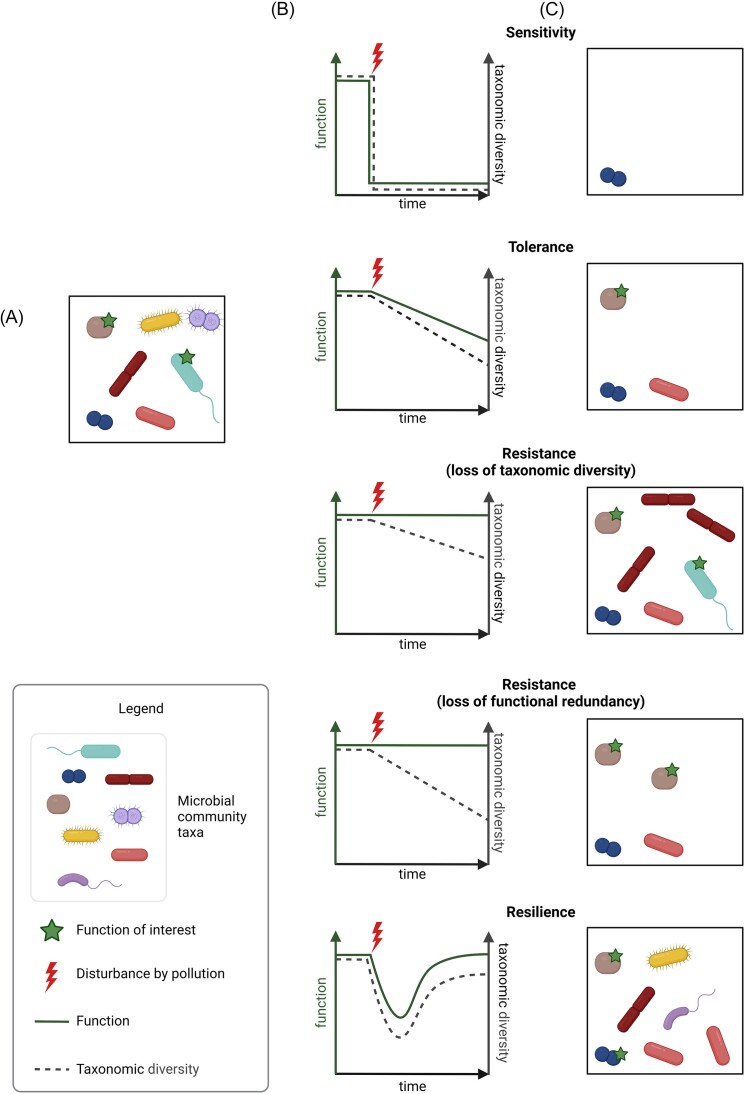
Possible outcomes in microbial community responses to pollutant disturbance with respect to a function of interest. (A) Model community initially composed of seven equally abundant taxa, some of which are capable of performing the function of interest (green star). This function may be directly associated with pollutant transformation, with another specific function (e.g. nitrogen fixation or nitrification), or with a widely distributed function (e.g. oxygen respiration). (B) Selected response profiles of microbial communities to disturbance (lightning) in terms of the function of interest (solid green line, left *y*-axis) and of taxonomic diversity (dashed black line, right *y*-axis). (C) Examples of microbial communities compatible with the different response profiles shown in (B) following pollution disturbance of the model community shown in (A). Apparent functional resistance to chemical pollution may involve loss of taxonomic diversity or functional redundancy, which will be detrimental for ecosystem functioning in the long-term. Functional resilience, i.e. the recovery of a particular microbially determined function following pollution disturbance, may feature changes in the taxonomic profile of the microbial community acting on the pollutant and gain of the functional ability to transform or degrade the pollutant by a resistant taxon through horizontal gene transfer, as shown. Of course, other community responses are also possible, such as gain of a degradation function upon pollution disturbance without apparent change of taxonomic diversity.

Microorganisms are essential players of natural ecosystems that cope with chemical and other environmental disturbances through the functions they perform (Delgado-Baquerizo et al. 2016, 2020, Cravo-Laureau et al. 2017, Borchert et al. 2021). The response of microbial communities to disturbances is intrinsically linked to their diversity (Allison and Martiny 2008, Tardy et al. 2014) (Fig. 2). For instance, more diverse communities provide greater functional redundancy (Birrer et al. 2017), thereby helping to maintain crucial functions even if the composition of the microbial community is altered (Herold et al. 2020, Walker et al. 2022). A major challenge faced by microbial ecotoxicologists and ecologists in polluted and pristine environments is linking taxonomic diversity to functionality. Although there is growing evidence that a loss in microbial diversity will inevitably lead to a loss of multifunctionality (Delgado-Baquerizo et al. 2016, 2020, Noyer et al. 2020, 2023), much work is needed to better understand the consequences for ecosystem services on a global scale. Thus, considering the response of microbial communities at different levels of taxonomic diversity and functional redundancy has strong potential to help us better understand the effects of chemical disturbances in the environment, as discussed in the following. Fortunately, molecular tools and approaches to do this in more detail are now increasingly available and are continuously being developed.

### The importance of tackling taxonomic diversity in microbial ecotoxicology

For both pristine and polluted environments, whether terrestrial, aquatic, or aerial, taxonomic alpha and beta diversities have been extensively studied in bacterial communities. Other types of microorganisms such as microeukaryotes (with the exception of diatoms in aquatic systems), archaea, viruses, and fungi remain less investigated, particularly in lotic and aerial ecosystems. Simultaneously studying alpha and beta diversities of several domains of life through metabarcoding can help to better understand how communities respond to disturbances (Delgado-Baquerizo et al. 2016, 2020, Noyer et al. 2020, 2023). For example, recent work on microbial communities of freshwater sediments experimentally exposed to copper has shown concomitant effects of the metal on the structure of bacterial communities (A-RISA method) and their functional potential. However, responses were variable over time during this chronic 21-day exposure: continuous effects during the experiment on some catabolic activities (β-glucosidase and phosphatase activities), resilience of other activities (denitrification and phosphatase activity), or time-lagged (respiration), while the bacterial structure remained impacted throughout the experiment. These results show the need for further study of these ecotoxicological processes on the diversity/function nexus, in particular the temporal dynamics of ecotoxicological effects (Mahamoud et al. 2018).

**Figure 3.**
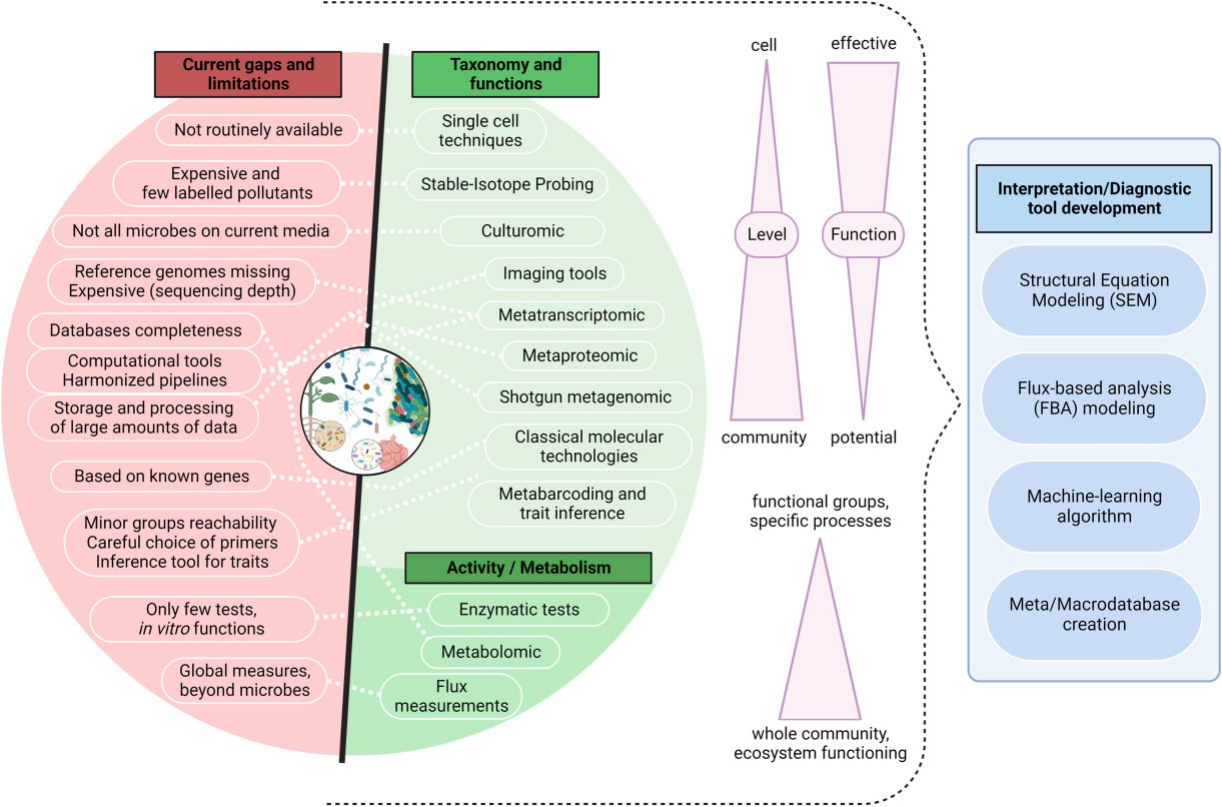
Overview of the current gaps and limitations of the main methods for studying microorganisms (diversity, activities) at different levels of complexity and perspectives for data interpretation and diagnostic tool development.

Environmental DNA (eDNA) metabarcoding enables to evaluate taxonomic diversity of bacteria or fungi, but rarely considers the whole microbial community. However, eDNA metabarcoding approaches are rapidly evolving, making it possible, e.g. to evaluate the diversity of microeukaryotes such as in marine environments impacted by offshore gas platforms (Cordier et al. 2019). Methodologically, nucleic acid (NA)-based approaches are the most widely used methods for high-throughput characterization of microbial communities at the taxonomic level (Fig. 3). Targeting DNA (who is present and potentially doing what) versus RNA (who is active now) will estimate different fractions of the community in a given environment and provide complementary information (Argudo et al. 2020). Many sets of ‘universal’ primer pairs targeting several domains have been designed to sequence amplicons and analyse microbial diversity. However, they are often biased against less dominant groups (Francioli et al. 2021, Tahon et al. 2021). Thus, carefully chosen domain-specific primers (e.g. Tahon et al. 2021, Tapolczai et al. 2021) remain the best available choice to provide detailed coverage of the taxonomic diversity of a domain of interest.

Identifying the roles and importance of every type of microorganism in any given polluted environment and under any physico-chemical condition is very challenging. Thus, microbial diversity in polluted environments is usually compared with that of reference pristine environments or along a pollutant gradient, or by monitoring changes in microbial composition before and after chemical disturbance. This approach has been applied in numerous studies in order to gain insights on the toxicity of pollutants and the resistance, resilience, tolerance, and adaptation of microbial communities to pollutants (Fig. 2; Morin et al. 2009, Lemmel et al. 2019a, Noyer et al. 2020, 2023). In so doing, certain taxa have been identified whose presence or absence in a polluted environment, or whose sensitivity to chemical exposure, showed potential as indicator species for use in ERA (Noyer et al. 2020, 2023, Lemmel et al. 2021, Bourhane et al. 2022, Veloso et al. 2023). This is further discussed in the dedicated section.

Nevertheless, comparisons of microbial diversity between different samples have several limitations. Some are technical and intrinsic to the applied methods (see Fig. 3) while others include the difficulty of securing reference pristine samples. This can be overcome using long-term observatories for environmental research. As an example, the SOERE PRO (Système d’Observation et d’expérimentation sur le long terme pour la Recherche en Environnement) dedicated to the study of organic residues in agriculture soils provides experimental devices to obtain long-term monitoring of the impact of pollutants associated with organic amendments compared with unamended sites. Another example of long-term microbial observatories is the International Long Term Ecological Research Network (ILTER) that counts 28 sites. In the same way, microbial ecotoxicology could benefit from longitudinal reference databases (Martínez Arbas et al. 2021; https://www6.inrae.fr/valor-pro_eng/French-Observatory-on-Organic-Residues/Objectives). However, discriminating against potential effects of other environmental parameters (as potential confounding factors) such as pH, temperature, or moisture in accounting for the observed changes in microbial communities exposed to pollutants remains a challenge.

### Effects of chemical disturbances on microbial functions—the need for a wider assessment

Directly measuring key ecosystem functions, when possible, is another way to assess the toxic effects of chemical pollutants on living microorganisms or communities (e.g. see review in Morin and Artigas 2023 for aquatic microbial communities). This can be achieved by directly monitoring processes *in situ* such as organic matter degradation by using litter bags (Lecerf et al. 2021) or microbial activities (respiration, enzymatic tests, photosynthesis, flux measurements, and so on) at the field scale (Bungau et al. 2021). Microbial respiration and denitrification have been widely used to account for chemical disturbances (Wakelin et al. 2013, Bérard et al. 2016, Lyautey et al. 2021) as well as other widespread enzyme activities such as urease, β-glucosidase, leucine aminopeptidase, acid phosphatase, and fluorescein diacetate hydrolysis activities (Fei et al. 2020, Lyautey et al. 2021, Li et al. 2022). The expression of activities from microbial functional guilds such as nitrifiers, which are less diverse functional groups, provides other highly relevant indicators to account for disturbances since their lower functional redundancy can lead to more deleterious consequences on ecosystem functioning (see dedicated section) (Simonin et al. 2016, Lu et al. 2022).

NA-based approaches also yield relevant data on microbial ecosystem functions and link the presence and even the expression of genes associated with chemical toxicity or pollutant degradation or transformation. Nevertheless, the same limitations as those mentioned in the previous section for taxonomy-associated genes apply. Moreover, in gene-specific PCR-based studies, readouts will be limited to genes with proven functional associations and related sequences that are amplified with the chosen PCR primers (Simonin et al. 2016). More generally, a significant knowledge gap remains regarding the impact of pollutants on microbial functions. More traditional molecular methods such as quantitative PCR (qPCR) and microarrays are also widely used to search for specific well-known functions such as hydrocarbon degradation (Yergeau et al. 2009). These approaches have also evolved with methods such as digital PCR, which was found to be appropriate to detect and quantify sequences of genes coding for the resistance to pollutants in biofilms (Kimbell et al. 2021). For a large proportion of the studies reported so far, investigated functions are directly linked to the pollutant of interest, such as genes involved in their degradation or transformation and corresponding metabolic pathways, especially if they are distributed across a wide range of microbial taxa. In such cases, the linkage between pollutants and functions and the associated taxa that accomplish them readily leads to the definition of new eukaryotic and/or prokaryotic indicators among enriched taxa in polluted environments. However, assessing the impact of new or emerging pollutants for which microbial responses are not yet well-investigated or understood is challenging. Yet we now have molecular tools that may help to decipher new metabolic pathways (see next section). Emphasis should now also extend to taxa or microbial groups poorly investigated or newly discovered that are involved in key ecosystem functions but not necessarily directly involved in the dissipation of chemical pollutants such as comammox and anammox bacteria. These bacteria are involved in a single-step production of nitrate from ammonium, and in the production of nitrogen gas from ammonium and nitrite (or nitrate), respectively (Li et al. 2021, Madeira and de Araújo 2021).

Fortunately, the increasing use of meta-omics approaches has begun to overcome the focus on well-characterized genes and pathways. Indeed, meta-omics make it possible to investigate, based on a unique shotgun sequencing experiment, the dynamics of all genes present or expressed in an environmental sample without *a priori* on the genes involved. Shotgun metagenomic sequencing (MGS) provides comprehensive information on the DNA present in a given sample, but requires extensive bioinformatic posteriori data analysis and is more expensive than previously mentioned methods (Ranjan et al. 2016, Douglas 2021). Advances in metagenome-assembled genomes (MAGs) technology, such as long-read sequencing and single-cell metagenomics, can improve the quality of MGS data. These technical advancements may lead to the discovery of specific genes responding to the presence of chemicals (Achermann et al. 2020) that once characterized functionally and tested through environmental ecotoxicology studies could become key bioindicators for microbial ecotoxicology. Nevertheless, methodology is not the only scientific bolt for studying diversity and function but also the way we connect it or not among life domains, which is very tricky, and the usual lack of quantitative estimation when using barcoding methods (relative and not absolute abundances).

More fundamentally, the lack of direct correspondence between taxonomic identity and a given function of interest limits the use of taxonomy-based investigations to characterize the microbial response to chemical pollution in microbial ecotoxicology. Moreover, work with DNA itself does not allow to gain insights into the physiological and metabolic state of microorganisms, while mRNA recovery remains challenging for some environmental samples and may limit the evaluation of *in situ* expression of microbial functions. Along the same lines, development of microbial metabolomics and increased knowledge of key metabolic pathways altered by responding to chemical pollution and of specific pollutant transformation pathways will help define new additional potential readouts for microbial ecotoxicology (Muller et al. 2018, Muller 2019) (see section entitled "Microbial roles in pollutant fate and transfer"). Gradually, this area will also benefit from new and ongoing advances in emerging experimental approaches (Malla et al. 2018) to help in environmental assessment and in the development of new remediation strategies (see the dedicated section).

In parallel, several recent initiatives aiming at developing an ecology-inspired conceptual framework to define microbial functional traits have emerged (Westoby et al. 2021). They are also intended to be used in the characterization of ecosystem functioning under different environmental conditions (Virta and Teittinen 2022), with applications for the toxicological assessment of chemical pollutants (Martini et al. 2021). Several easy-to-use tools or databases providing potential functional information such as PICRUSt2 (Douglas et al. 2020), Tax4Fun (Asshauer et al. 2015), bactoTraits (Cébron et al. 2021 for bacteria), FUNGuild (Nguyen et al. 2016), and FungalTraits (Põlme et al. 2020 for fungi) have been reported for this purpose (see Fig. 3). They can help assign functions or traits based on taxonomic identities and help identify bioindicators for ERA. However, the inference of functionality from taxonomic diversity remains challenging, particularly concerning pollutant degradation. Indeed, it is common that within the same bacterial species, some strains can degrade or transform and others not. Moreover, the lack of functionally characterized reference microorganisms of known genomic sequences, as well as the large proportion of genes with unknown functions in sequence databases, still limits the application of such tools for robust prediction of ecosystem functioning from taxonomic diversity. Although the number of available traits is still limited, it will be progressively enriched with ongoing progress in this area, in particular for the large number of taxa for which a corresponding set of traits usable for environmental assessment is still lacking. Some biases also persist due to the fact that these tools are generally more extensively developed for bacteria than other microorganisms such as fungi or algae (Berg et al. 2020, Douglas 2021). The use of microarrays (e.g. Geochips; He et al. 2010) allowing to target thousands of functional genes could help in identifying which functions are impacted by pollutants (He et al. 2012). Recently, a new general and more powerful generation of biochip has been introduced in order to link microbial genes/populations to ecosystem functions (Shi et al. 2019).

### Applying fundamental concepts in ecology to microbial ecotoxicology—potential benefits

Excitingly, the emerging renewed emphasis on analysis of ecosystem functioning fuelled by functional genomic approaches now enables us to apply classical fundamental questions and concepts of macroecology to the microbial compartment (Muller 2019). This seems of particular relevance for microbial ecotoxicology. Indeed, key issues in the assessment of tolerance, resistance, or adaptation of ecosystems to chemical stress, and of their resilience, can now be addressed for the microbial compartment as well. For the characterization of ecosystem functions, it is now possible to investigate the relevance not only of the presence or absence of specific genes or taxa but also of the co-occurrence or even of the interactions of specific sets of genes and/or taxa and their dynamics upon exposure to chemicals. While this research area is still in its infancy, several important studies have recently been reported, and display the great potential of the corresponding findings as bioindication tools for microbial ecotoxicology. Emerging initiatives using ecology-inspired approaches (Virta et al 2020a) and omics (for review see Seneviratne et al. 2020), such as the combination of metagenomics, metatranscriptomics, metaproteomics, and metabolomics, provide new insights into the functional networks that arise, e.g. following pollution or during biodegradation of pollutants (Muller et al. 2018). For instance, Herold et al., (2020) demonstrated through a multi-omics approach that resistance and resilience properties of wastewater treatment plant communities to a disturbance depended on phenotypic plasticity and niche complementarity.

On the other hand, because not every ecosystem function is favoured by higher community diversity, the combination of complementary experimental approaches, including omics (see Fig. 3), together with appropriate statistical or machine learning methods may allow accurate assessment of changes in alpha diversity along with the underlying stochastic–deterministic assembly processes. Recent advances in machine deep learning approaches (e.g. using random forest or deep convolutional neural networks) can help to elucidate relationships between the composition of the microbiome and its functions or to monitor changes in the composition of the microbiome in response to environmental stresses (Hernández Medina et al. 2022). Deep learning can also be applied for image analyses to study the morphometry of microbial taxa and is being developed for diatoms, algae, fungi, and bacteria (Kloster et al. 2020, Picek et al. 2022, Xu et al. 2022, Venkataramanan et al. 2023). These tools are complementary to molecular biology approaches and morphology-based taxonomy in microbial ecotoxicology studies, due to their potential to characterize the effects and fate of pollutants at the ecosystem scale and the taxonomic, behavioural, and morphometric responses of microbial communities (in particular protists and microalgae) to pollutants. Also noteworthy is the recent development of image analysis and spectral imaging tools (associated even more recently with deep learning) to study changes in ecosystems (e.g. remote sensing applied to aquatic environmental monitoring; Li et al. 2020, Sagan et al. 2020). Finally, the development of mechanistic computational models to analyse the dynamics of complex microbial interactions at different levels is also promising (Henry et al. 2016, Niarakis and Helikar 2021).

In this way, both taxonomic and functional knowledge of microbial communities in an environment of interest may inform on the nature and extent of toxic effects of pollutants, and on the capacity of the ecosystem to functionally recover from pollutant exposure (Fig. 2). Such a combination of experimental and advanced analytical methods was found essential to understand the impact of pollutant disturbance on complex microbial communities in activated sludge bioreactors (Santillan et al. 2019) and on bacterial diversity along a river-to-estuary gradient (Meziti et al. 2016). Notably, pollutants may adversely affect microbial functional and phylogenetic diversity through cascading effects on biochemical processes (Meena et al. 2020). While likely very significant, the effects of multiple ecological interactions and associated unsuspected links between diversity and function are still rarely described.

The use of structural equation modelling (SEM; Xiao et al. 2021), machine-learning algorithms and metabolic models, such as Flux-Based Analysis (FBA; Cuevas et al. 2016), may provide a cumulative understanding of the direct effects of pollutants on microbial diversity and functions, as well as of the cascading effects between interacting organisms in an holobiont or in an ecosystem. These approaches may be useful to test and evaluate multivariate causal relationships (Fan et al. 2016), as shown by Simonin et al. (2016). SEM has also already been combined with machine-learning algorithms (particularly random forests) to provide insight into the direct and indirect effects of different stressors and diversity on the ecosystem multifunctionality (Delgado-Baquerizo et al. 2016, 2020).

## Microbial roles in pollutant fate and transfer

The chronic or repeated exposure of microorganisms to chemical pollutants can lead them to develop direct and indirect metabolic or detoxification pathways to degrade, transform, or accumulate them. Degradation or transformation of pollutants can be considered as an ecological function beneficial for the environment contributing to reduce their persistence and consequently, exposure and toxicity towards living organisms. Microbial activities can also affect pollution fate by releasing toxic elements from their carrier phases e.g. release of arsenic or mercury through redox reactions and mineral solubilization (Hellal et al. 2015, Héry et al. 2015). Unfortunately, some microbial activities can lead to the formation of compounds with greater toxicity [e.g. perchloroethene (PCE) to 1,2-dichloroethene (DCE), and vinyl chloride (VC); Adrian and Löffler 2016]. With the continual emergence of new synthetic chemicals and the remaining knowledge gaps about historical ones, many questions remain on the roles of microorganisms in pollutant fate and transfer. In particular, there is a need to identify the major microbial actors involved in the degradation and transformation of synthetic chemicals *in situ*. Innovative approaches based on holistic, multidisciplinary, and integrating new technologies (Fig. 3) are also needed to cope with the complexity of interactions between microbes, and between microbes and their environment (biotic and abiotic factors) and pollutants.

### Pollutants as a selective force

The range of currently known chemical pollutants, whether natural (metals and metalloids, hydrocarbons) or anthropogenic (pesticides, plastics, pharmaceuticals, etc) is extremely vast and in continuous expansion due to the constant release of new molecules and the production of a myriad of intermediate degradation metabolites. Although there are many studies on their transformation or degradation (Parales and Haddock 2004, Gadd 2010, Duran and Cravo-Laureau 2016, Hidalgo et al. 2020) there are still many knowledge gaps to be filled on the microbial mechanisms involved in these reactions.

**Figure 4.**
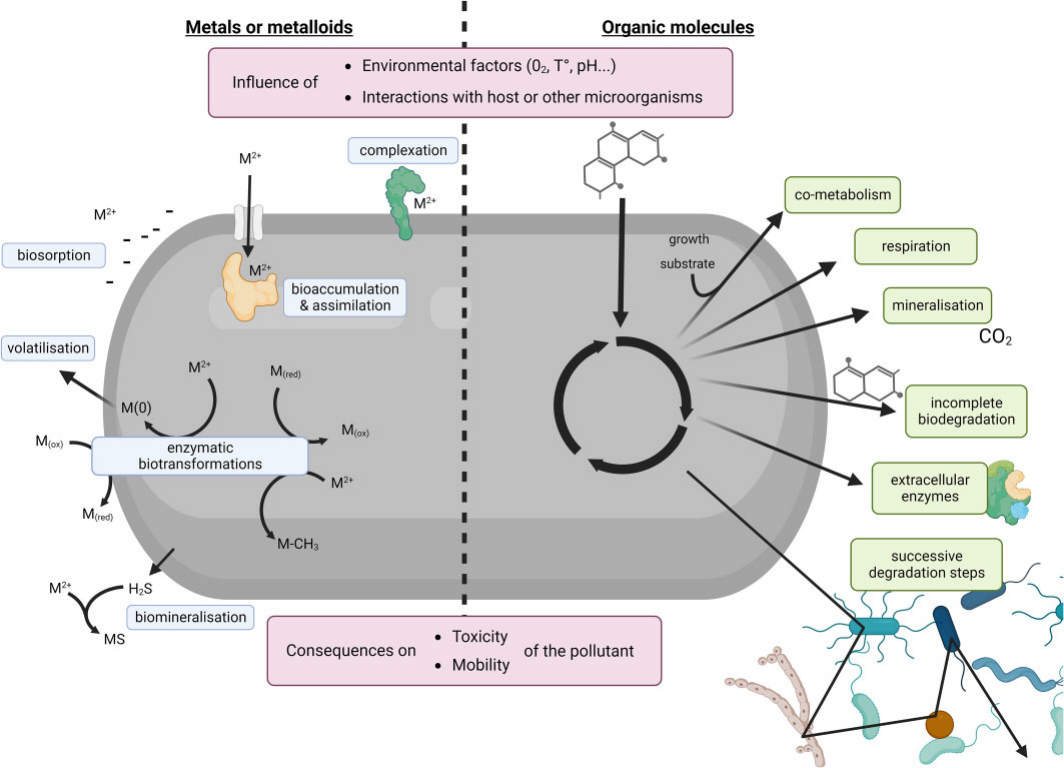
An overview of microbial transformation and degradation mechanisms of metals and metalloids (left) and organic molecules (right).

Metallic compounds can accumulate in the environment due to human activities (e.g. Cu and Zn in agriculture, Hg and As in mining, and so on). They can be biotransformed by enzymatic reactions (oxidation, reduction, methylation) (Fig. 4). These reactions can be a defensive mechanism (e.g. Hg reduction encoded by the *mer* operon; Barkay et al. 2003) or a consequence of metabolic activity (e.g. As(V) or Fe(III) reduction). Apart from these well-known examples, microbial interactions with metals or metalloids of emerging concern remain poorly documented. For example, knowledge on the uptake, efflux, and redox transformation pathways of antimony is limited (Deng et al. 2021). Recently, new practices have led to an increase in the use of rare earth elements and metal nanoparticles in different sectors (e.g. agriculture, remediation, cosmetics, batteries, and so on). However, the role of microorganisms on the fate of these elements in the environment remains poorly understood (Xie et al. 2017, Crampon et al. 2018, Eymard-Vernain et al. 2018).

For many of the ever-expanding range of organic pollutants, there is no data on their biodegradability or transformation potential. Many organic pollutants can be directly transformed by microorganisms and are used as a carbon source (e.g. polycyclic aromatic hydrocarbons (PAH) degradation) and/or as electron donors or acceptors (e.g. organohalid respiration of chloroethenes). They can also be transformed indirectly through cometabolic reactions (e.g. chloroethenes; Dolinová et al. 2016, Zhang et al. 2019) (Fig. 4). Many degradation pathways and the corresponding genes are well-characterized, yet microbes degrading (new) emerging organic compounds need to be identified and their metabolic pathways further studied in order to develop adapted remediation strategies. The use of next-generation physiology approaches that are independent from *a priori* knowledge of genomic information could allow to focus on cellular functions (Hatzenpichler et al. 2020). These approaches combine microbial phenotype probing, high throughput cell sorting, and downstream techniques such as single-cell sequencing, targeted cultivation (e.g. culturomics; (Martiny 2019, Almeida et al. 2022), or complementary microscopy or imaging analyses (Fig. 3). A change of perspective is also required to favour understanding of *in situ* situations rather than of pure strains exposed to one compound (or family of compounds). Indeed, degradation can involve microbes interacting in a consortium (e.g. syntrophy; Thomas et al. 2019) and many other biotic and abiotic factors can simultaneously influence degradation (Chishti et al. 2021, Yuan et al. 2021). Understanding *in situ* reactions will contribute to identifying ERA indicators and adapting bioremediation to the environmental context.

An emerging topic related to microorganism-pollutant interactions is the occurrence of increasingly complex associations of different compounds and their consequences for microbial communities and their transformation potential. Phenotypic probing approaches such as SIP (stable isotope probing), will allow the identification of microorganisms actively involved in specific metabolic processes such as the degradation of organic pollutants (Lemmel et al. 2019b). A general effort has also been made in recent years to improve isolation of microbial strains from understudied taxa (Chaudhary et al. 2019), (since this remains the approach of choice to study their metabolism and particularly their role in the transformation of pollutants) and potentially use them for bioremediation purposes (Fig. 3). However, it is beginning to be possible to predict the modes of action, effects, behaviour, and transformation of pollutants *in silico* (Han et al. 2019, Singh et al. 2021). This type of approach would make it possible to assess ecotoxicity and fate of new molecules more quickly and comprehensively, even if the prediction of effects of pollutant mixtures remains a challenge.

### A complex network of microbial interactions and collaborations

Although many studies have been carried out on single microorganisms exposed to a particular pollutant, holistic approaches are now needed to better understand the different levels and means of biotic interactions. Indeed, biotic interactions can occur within a domain or between domains and also include trophic interactions or host–microbiome interactions (Adamovsky et al. 2018) (see dedicated section). Unravelling these complex interactions *in situ* is a major challenge for microbial ecotoxicology. For example, positive interactions between fungi and bacteria have recently been demonstrated (Álvarez-Barragán et al. 2022) where bacteria can be dispersed in PAH-polluted environments via fungal hyphae, allowing to overcome barriers and promote accessibility to PAHs. Other examples are the cascades of redox conditions that lead to the dehalogenation of PCE to ethylene by promoting optimal conditions for halorespiring bacteria such as *Dehalococcoides* sp. (Hellal et al. 2021), or the total degradation of PAH by a microbial consortium in successive degradation steps (Thomas et al. 2019) (Fig. 4).

Biodegradation and transformation reactions are also tightly controlled by environmental factors. A better understanding of how environmental, physico-chemical, and operational (in a bioremediation context) parameters drive microbial diversity and activity is required to develop effective and robust bioremediation strategies (Laroche et al. 2018), as well as how it impacts pollutant bioavailability and speciation (Barral-Fraga et al. 2020). In a context of global change, this reinforces the importance of combining laboratory and *in situ* approaches for more realistic conditions and ecological relevance, and of developing models for biogeochemical processes allowing to disentangle between correlation and causality.

### Consequences on pollutant behaviour and transfer

Microbial activity can impact the mobility of metals and metalloids through the dissolution or the precipitation of metal-bearing minerals (Dong et al. 2022). Dissolution of metal-bearing minerals will contribute to impacting previously pristine environments. Conversely, the immobilization of toxic elements by precipitation or adsorption results in natural attenuation of the pollution (Egal et al. 2010). This is of particular importance in continuums (soil/coastal marine environments) or at the interface between different conditions (oxic/anoxic) or compartments (water/sediments) (Héry et al. 2014, Hellal et al. 2015, Zhang et al. 2020). Pollutants as well as microorganisms can also be transferred from one environment to another (Châtillon et al. 2023). For example, adsorption of metals to the surface of microplastics (Liu et al. 2021) can impact their fate since microplastics act as vectors of metallic pollutants and attached microorganisms towards aquatic environments or organisms (Wang et al. 2021). Recently, it has been suggested that such complex interactions may also promote the transport and diffusion of antibiotic-resistance genes in the aquatic environment (Marathe and Bank 2022).

### Specificities of experimental microbial ecotoxicology in deciphering the role of microorganisms in pollutant biotransformation and transfer

Current knowledge gaps on biotransformation processes under controlled laboratory conditions or under environmentally relevant conditions lead microbial ecotoxicologists to innovate at the experimental level. Simplified microbial experimental systems have been particularly useful to address ecological questions allowing for experimental controls (see reviews by Jessup et al. 2004, 2005, Cravo-Laureau and Duran 2014). Future research should now reach beyond these relatively simple models and attempt to address the complexity of the realworld. This issue of upscaling is a major challenge (Bonnineau et al. 2021, Guasch et al. 2022). Transdisciplinarity has always been central in microbial ecotoxicology and is now also taking on board new technologies in chemistry and biology, in particular for investigations at different scales, dynamics and levels of complexity, in order to improve our understanding of the fate and transfer of pollutants in the environment (Fig.   3). In the future, identifying and referencing the degradation or biotransformation pathways of pollutants and products/metabolites will be essential to better understand all the chemical entities (exposome) presented to microbial communities in a situation of interest. An ideal microbial ecotoxicology database should be comprehensive, interdisciplinary, and multiscale, and include data on microbial diversity and functions, metabolites, metabolic pathways, physico-chemical conditions, chemicals, and pollutants. However, much work remains to be done to develop these databases and make them usable.

Being able to estimate the contribution of microbial communities to the transformation and fate of toxic compounds will allow a better estimation of the persistence of pollutants (half-life, dT50) in natural environments (see dedicated section). The identification of families of compounds and chemical structures that are more easily degraded by microbial communities or less likely to be bioaccumulated (and thus transferred through the trophic chain) will help provide guidelines for the design of new green chemicals that should have a reduced impact on ecosystems. A better understanding of the mechanisms involved in the interactions between microorganisms and pollutants is thus a prerequisite for the development of effective and sustainable bioremediation strategies in the future (see dedicated section).

## Linking impacts on microbial communities to impacts and risks for ecosystem and host functioning

**Figure 5.**
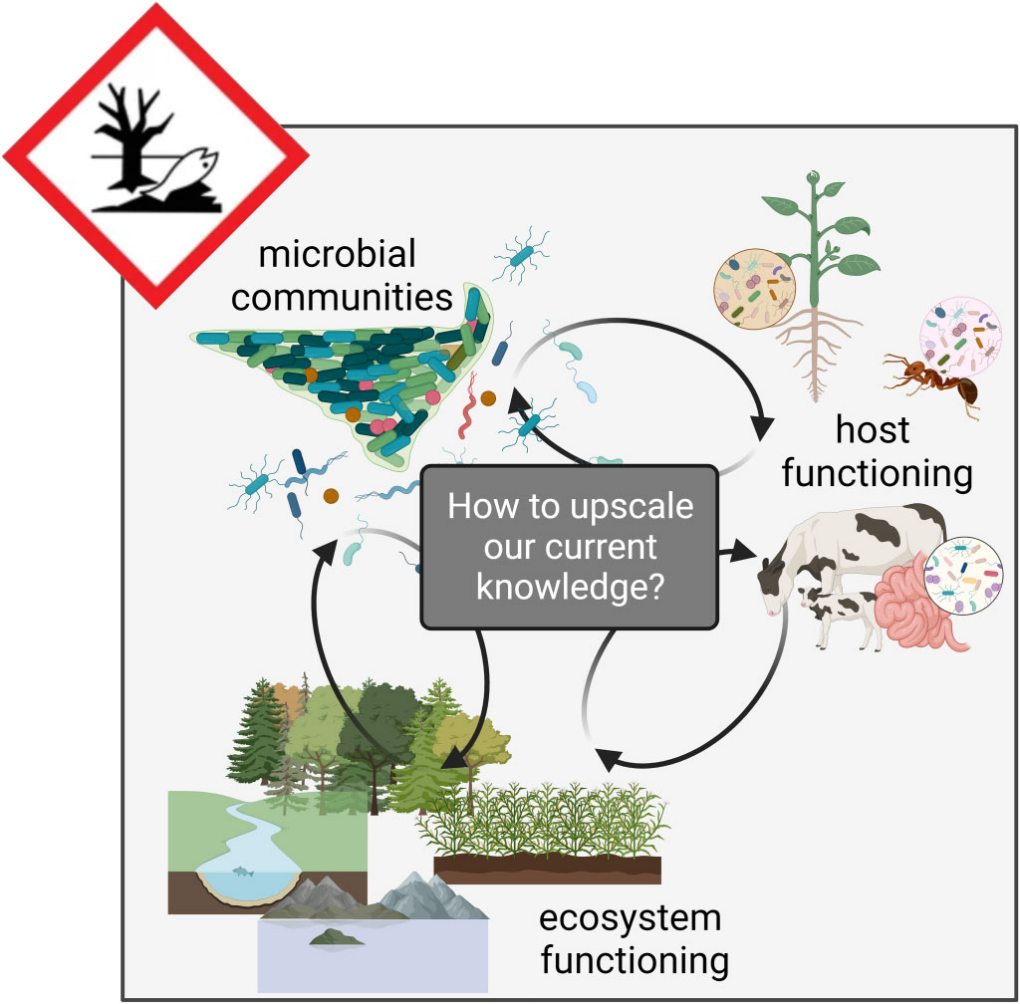
Illustration of the current paradigm shift and how impacts of pollutants on microbial community diversity and functions can in turn affect host and ecosystem functioning.

As illustrated in the first section, important conceptual and methodological advances have been achieved in the last decades to assess the effects of pollutants on microbial diversity and functions in polluted ecosystems (Pesce et al. 2020b, Morin and Artigas 2023). These advances have also made it possible to study pollutant effects on the interactions occurring between various animal or plant organisms and symbiotic microorganisms, including e.g. microbiomes (Duperron et al. 2020) and rhizosphere microbial communities (Barra Caracciolo and Terenzi 2021). Yet some authors recognize the importance of microbial ecotoxicology research in other fields such as animal conservation biology (Trevelline et al. 2019) or human and animal health (Adamovsky et al. 2018, Greenspan et al. 2022) underlining the importance of studying the links between microbial communities and their hosts (Fig. 5).

### A lack of knowledge due to the difficulty of assessing complex interactions

Knowledge of the consequences of ecotoxicological effects on microbial communities at the scale of ecosystems or symbiotic partners is still scarce. As an exception, many studies have dealt with the effects of pollutants on the interactions between microorganisms and plants. However, these studies mainly aimed to improve agronomic practices (e.g. selection of plant growth-promoting rhizobacteria strains resistant to pesticides, for inoculation in conventional agriculture and compensation of the inhibition of natural symbioses; Ahemad and Khan 2010), or phytoremediation (e.g. use of microorganisms to improve the uptake capacity of plants for metals; Yang et al. 2022). Yet, there is still a lack of studies assessing this kind of interaction in an ecotoxicological framework.

This limited knowledge is primarily explained by the fact that concepts and methods in microbial ecology for linking microbial communities to ecosystem functions are still in their infancy (Orland et al. 2019, Morris et al. 2020, Codello et al. 2023). Despite this, it is now well-recognized that microbiomes are affected by the same threats as their hosts, with environmental pollution among the most important (Trevelline et al. 2019). First, pollution alters the composition of environmental microbial communities (see previous sections) from which the host can build up its microbiome. Second, pollutant toxicity can also directly alter the host-associated microbiomes by increasing resistance (Lapanje et al. 2010) or tolerance (Costa et al. 2016) to pollutants, or by decreasing microbial diversity and, consequently, causing the loss of functions (Kakumanu et al. 2016) potentially important to the host (Fig. 2). Third, host microbiomes can respond to pollution by transforming pollutants into more toxic metabolites affecting the host (Pinyayev et al. 2011, Claus et al. 2016). Moreover, several studies suggest that a loss of microbial diversity (Tardy et al. 2014, Delgado-Baquerizo et al. 2016, 2020, Laforest-Lapointe et al. 2017) or microbial interactions (Wagg et al. 2019) can impair ecosystem multifunctionality. However, examining the relationships between microbial community structure and ecosystem (Graham et al. 2016) or host (Adamovsky et al. 2018, Duperron et al. 2020) functioning remains challenging. This is first due to the existence of high functional redundancy within microbial communities including gut microbiota (Moya and Ferrer 2016). Of particular interest in this regard is Allison and Martiny’s (2008) conceptual approach to how disturbances may or may not alter ecosystem processes through microbial functions. Their model is based on levels and patterns of functional redundancy and made clear the lack of data on the links between microbial phylogeny, physiological traits, and responses to disturbance. Second, it is extremely difficult if not impossible to have a reference point of the pristine environment or holobiont, with which to perform comparisons in an ecotoxicological context. When studying host-sheltered communities, the lack of pristine habitat can translate into lack of knowledge about what a eubiotic (versus dysbiotic) host-associated microbiome is.

### Upscaling in microbial ecotoxicology: limits, pitfalls, and possible solutions

Besides the difficulties in extrapolating microbial ecotoxicological responses to the ecosystem scale, it is important to emphasize that ecosystem processes and functions are not only driven by microorganisms but also by abiotic factors and/or by biological processes carried out by macroorganisms (van der Plas 2019). Indeed, a potential limitation of up-scaling from microbial ecotoxicology is that it may be difficult to accurately predict the effects of toxic chemicals on ecosystems or holobionts (Duperron et al. 2020) based solely on their effects on microorganisms. Different classifications of ecosystem functions are available in the literature (e.g. Pettorelli et al. 2018, Garland et al. 2021, Pesce et al. 2023) and most of them involve microorganisms which are sometimes the major contributors. One of the best examples is the prominent role of microorganisms in nutrient cycling (Garland et al. 2021). The effects of chemical pollutants on the capabilities of microorganisms to contribute to nutrient cycling are widely studied in soil and aquatic environments using a combination of various approaches (from molecular to potential or effective activity measurements; see Fig. 3). However, these assessments are generally carried out in small-scale laboratory studies or at the scale of microhabitats. This severely limits the possibility of upscaling to the ecosystem level, especially if complex interactions with key environmental and/or nonmicrobial biological factors are not taken into consideration (van der Plas 2019), and lead to unpredictable cascading effects. Besides the issue of spatial heterogeneity, temporality also needs to be taken into consideration depending on the capacity (or not) of microbial communities to cope with chemical and other environmental stresses (e.g. according to their adaptation, resistance, and resilience capacities; Allison and Martiny 2008; Fig. 2). Moreover, it is important to note that some categories of functions are not or only minimally considered in microbial ecotoxicology even though they strongly involve microorganisms (e.g. soil/sediment formation or erosion; based on the classification proposed by Pettorelli et al. 2018). Thus, assessing the impact of pollutants on the functioning of ecosystems through the prism of the response of microbial communities to these chemicals probably requires a paradigm shift. Indeed, rather than addressing this issue primarily from a microbial perspective, it would be relevant to approach it from the perspective of ecosystem function by determining the most relevant study scale. Thus, taking the above example of nutrient cycling, it seems necessary to develop approaches that combine measurements at the scale of microbial communities (exoenzyme production, catabolic activities, and so on) with others carried out at the scale of ecosystems such as measurements of nutrient flows. From this point of view, the ecosystem services approach of Hayes et al., (2018) based on logic chains (linking direct ecotoxic impacts, via secondary interactions, to impacts on ecosystem processes/properties) seems promising for targeting the areas of research to be developed in order to better quantify the effects of pollutants on ecosystem services. Hence, in their case study (i.e. responses of ecosystems to soil copper pollution), the authors emphasized the need to focus on several microbial activities (i.e. organic matter decomposition and nutrient cycling) to take into account the role of microbial communities in ecosystem functions (Hayes et al. 2018). These shifts in scale in relation to an ecosystem function perspective suggest that it is necessary to manage these microbial ecotoxicology issues through a strong interdisciplinary approach. For example, the above-mentioned ecosystem function of erosion limitation, which is likely to be impacted by pollutants, requires the combination of microbial ecotoxicology measurements with physical measurements (Gerbersdorf et al. 2005, Crouzet et al. 2019).

As mentioned above, microbial communities, being an integral part of the ecosystem, are subject to other factors than chemical pollutants. We therefore highlight the importance of taking multistress into account in microbial ecotoxicology studies, reflecting not only the reality of pollutant mixtures but also the reality of climate change (Zandalinas et al. 2021). This latter high-stakes subject is beginning to be considered by the scientific community (Luo et al. 2021, Courcoul et al. 2022) and recent work is also attempting to address this issue by integrating microorganisms into different ecosystem levels (O’Brien et al. 2022, Vijayaraj et al. 2022).

## Microorganisms as a tool for environmental risk assessment and bioremediation

**Figure 6.**
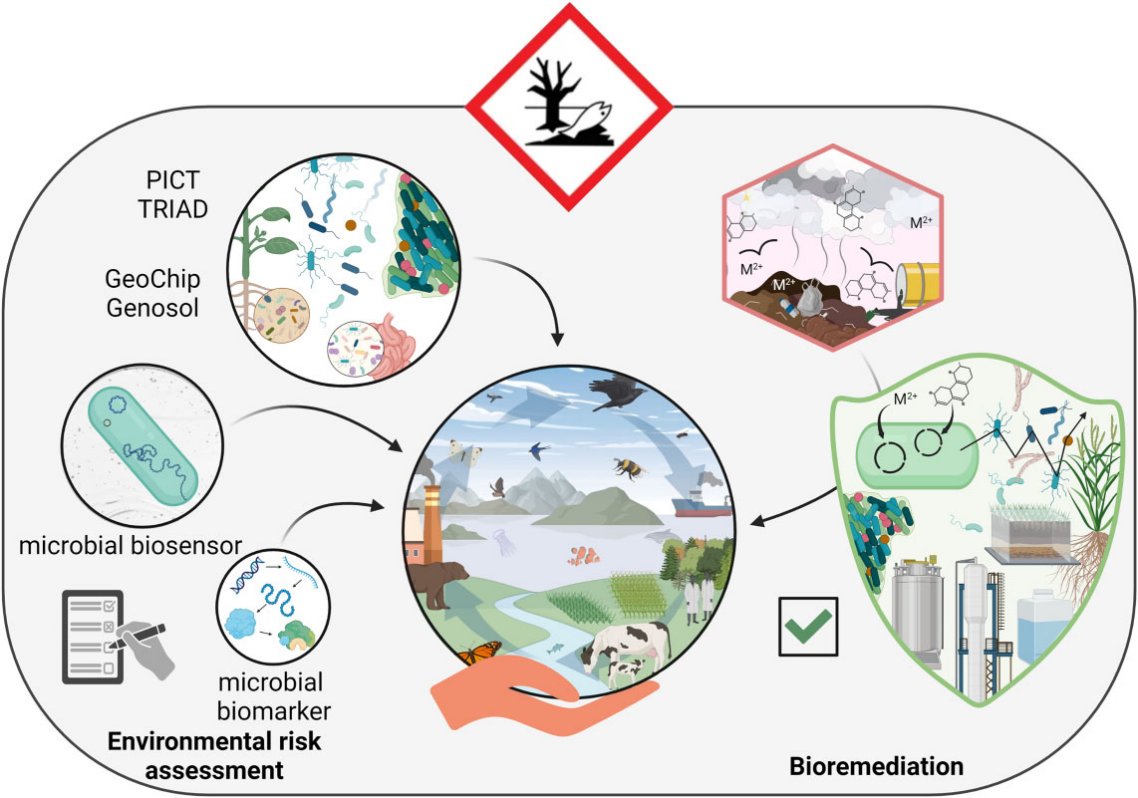
Illustration of different applications of microorganisms as tools for ERA and bioremediation.

This section aims to illustrate the range of existing applications of microbial ecotoxicology where microorganisms are used as tools for ERA (limiting the scope to EU for standardized and normalized tests) and bioremediation, what potentially limits their application and where future developments and opportunities lie (Fig. 6).

### Microorganisms for ERA

Despite the recognized importance of microbial communities in numerous ecological functions supporting ecosystem services and a large number of reported microbial-based methods (Bouchez et al. 2016), only a few tools based on microorganisms have been standardized and used for ERA (Table 1). Some of them, such as the single-species tests Ames, Microtox, or the microalgal test are mainly used for *a priori* ERA, to predict hazards and assess risks before a new active compound is brought onto the market. Others such as the Biological Diatom Index are applied for *a posteriori* ERA to assess the ecotoxicological impacts of chemical residues in the environment. Complementary approaches have to be applied to integrate microorganisms from aquatic and soil ecosystems into ERA (Escher et al. 2023).

**Table 1.** Some existing tools based on microbial ecotoxicology concept for ERA, presented by context of application.

Tool name	Microorganism or DNA exposed	Characteristics	Application	Reference
*A priori* risk assessment				
Ames	*Salmonella typhimurium* strains His negative	Mutagenic potential of a compound	Toxicity evaluation during the development of new compounds.Toxicity analysis of samples (urines)	OECD 471 (OECD 2020)
Microtox	*Vibrio fischeri*	Bioluminescence inhibition of a compound.	Toxicity evaluation during the development of new compounds Water quality evaluation	ISO 11348
Microalgal test	*Raphidocelis subcapitata*	Growth or photosynthesis inhibition of a compound.	Toxicity evaluation during the development of new compoundsWater quality evaluation	ISO 8692
Mycorrhizal fungi test	*Glomus mosseae*	Fungal spore germination inhibition of a compound	Toxicity evaluation during the development of new compounds Soil quality evaluation	ISO 10832
***A posteriori* risk assessment**				
Pollution-induced community tolerance (PICT)	Phototrophic and heterotrophic microbial communities	Need for a community from an uncontaminated environment considered as the control.	Diagnostic and risk assessment tool for aquatic environment. Transferable to sediments and soil.	Blank et al. (1988)
Triad	Soil microbial community Not exhaustive	Combining three data sources (chemistry, ecotoxicology, and ecology).	Ecological risk assessment specific to contaminated sites and soils. Transferable to other environments	ISO 19204
Diatoms Biological Index (DBI)	Diatoms	Morphological analysis requiring in-depth taxonomic knowledge and expertise	Used in the European Water Framework Directive for river ecological assessment	Prygiel, and Caste (1998).

#### A priori *ERA: illustration with the case of pesticides*

In Europe, the *a priori* ERA of active ingredients in pesticides is conducted in compliance with the 1107/2009/EC directive, which authorizes market delivery. For soil microorganisms, ERA of active ingredients solely relies on the assessment of their effects on nitrogen (OECD 216; OECD 2000a ) and carbon (OECD 217; OECD 2000b) mineralization. In order to better protect soil ecosystem services, EFSA (European Food Safety Authority 2013) proposed to set up a series of specific protection goals including the protection of functional groups of microorganisms. Seven years later EFSA (2017) proposed a set of endpoints to be considered for the protection of in-soil living organisms, including nitrifiers, a microbial guild involved in the N-cycle (Ockleford et al. 2017), and arbuscular mycorrhiza fungi (AMF), which form an obligate symbiosis with most higher plants. However, despite these two scientific opinions (EFSA 2013, 2017), specific protection goals and criteria have not yet been implemented in the ERA of active ingredients. One reason for this is that the endpoints proposed to fulfill the specific protection goals of key soil ecological functions are criticized (Sweeney et al. 2022), with some authors pointing out the difficulties in concluding on the origin of observed effects (direct or indirect) on these endpoints after pesticide exposure (Karpouzas et al. 2014).

In addition, there is still a need for research to better define effect thresholds based on the acquisition of the normal operating range (NOR) of each microbial endpoint in order to consider their possible recovery following the dissipation of the active ingredients and of their degradation products (Brock et al. 2018). The interactions that microorganisms have among them and with their host were recently shown to have an impact on pollutants fate (cometabolism) and toxicity. In addition, it is now time to move from *a priori* assessment of the effect of a single pure active ingredient on a single species or function to *a posteriori* assessment of complex environmental situations where complex mixtures of chemical residues of different origins are often found. To this end, microbial biosensors, defined as analytical devices combining living microorganisms as a sensing element with a process of integration of the metabolic or physiological state through a transducer, could be suitable to tackle this challenge. The toxicity of several organic and inorganic pollutants has been investigated using either microbial cell fuel biosensors, associated with microbes from activated sludge, biofilm, or specific species (Dávila et al. 2011, Zhou et al. 2017, Uria et al. 2020), or reporter biosensors, using recombinant microbial strains (Jia et al. 2012, Durand et al. 2016).

#### A posteriori ERA

Integrative methods are required to diagnose *in situ* toxicity in order to improve the *a posteriori* ERA of various pollutants (Table 1). The PICT approach is recognized as a relevant method to demonstrate the direct causality between environmental pollutant pressure and *in situ* response of microbial communities in both aquatic (Tlili et al. 2016) or soil environments (Campillo-Cora et al. 2021). However, the application of PICT still faces several challenges that deserve further investigation. First, establishing the NOR of tolerance of microbial communities is one main issue requiring large reference datasets that remains poorly tackled (but see Blanck et al. 2003, Campillo-Cora et al. 2021). Second, there is a need to develop and validate *via* ring-testing standardized protocols based on published methods (from *in situ* sampling to modelling and interpretation of lab toxicity test results). Third, new methods are required to increase the diversity of considered pollutants and microbial functions used as endpoints in the toxicity tests to evaluate tolerance levels. Fourth, guidelines need to be elaborated to interpret community tolerance with reference to the tolerance baseline (Campillo-Cora et al. 2021). Finally, new knowledge should be acquired to understand the processes involved in PICT responses (influence of confounding factors, cotolerance processes, etc; Tlili et al. 2016). In addition, the costs of adaptation of microbial communities to pollutants (e.g. secondary effects on microbial diversity and ecological functions; Tlili et al. 2011, Bérard et al. 2016, Pesce et al. 2020b) should be studied to give clues on how to transform PICT responses into an assessment of ecological risks and ecotoxicological effects at the microbial community and ecosystem levels.

The Triad approach, which combines toxicity testing and chemical and ecological data of a site to determine the effect of pollution on the ecosystem, can also be viewed as a promising tool (Gutiérrez et al. 2015, Klimkowicz-Pawlas et al. 2019). Although this ISO standard lists several standardized methods for measuring each of the identified risk types, the use of nonstandardized methods can be employed. Due to their ubiquity and different ecological roles in the environment, microbial communities could constitute good indicators to consider in this approach.

Currently, only microalgae are considered in the EU Water Framework Directive for the calculation of indices based on diatoms and phytoplankton. Although these indices have gained importance for the assessment of the ecological quality of aquatic ecosystems (Venkatachalapathy and Karthikeyan 2015, Lavoie et al. 2018), their output remains poorly informative with regards to the ecological effects of a large variety of pollutants. Such indices could be further developed to take into account the impacts of pollutants. In combination with relevant pollutant monitoring studies, the increasing application of diatom DNA metabarcoding will help to monitor the effects of pollutants on diatoms (Tapolczai et al. 2019, Maitland et al. 2020). This is likely to be also applicable to other microbial groups and in different types of ecosystems and environmental compartments due to the concomitant and ongoing improvement of sampling and analytical methods for characterizing environmental pollution by a large variety of chemicals (Hollender et al. 2017) and of eDNA studies (Seymour 2019). A few years ago, Bouchez et al. (2016) defined the level of operability of several molecular microbial indicators according to each environmental matrix (i.e. soil, sediments, water, atmosphere, and wastes) for environmental diagnosis. For the ERA of herbicides in soil, some authors suggest searching for tolerant and/or sensitive populations of nontarget microorganisms that nonetheless carry the enzyme specifically targeted by the active ingredient (Petric et al. 2016, Thiour-Mauprivez et al. 2019). Monitoring the quality of aquatic environments could also be done by integrating bacteria naturally present in the aquatic compartment to propose a new generation of microbial biosensors (Zhou et al. 2017, Jiang et al. 2018, Fang et al. 2020).

### Microorganisms as tools for assessing ecosystem functioning

Ensuring environmental and human health protection requires preserving or restoring ecosystem functioning and their capabilities to provide services. As noted previously, the ubiquity of microorganisms and the numerous ecological functions they perform make them essential key drivers that must be protected in order to ensure the continued functioning of ecosystems and preserve the One Health concept. In 2023, an EU directive on soil protection is on the verge of being adopted by the European Commission 17 years after its first proposal. In the meantime, several EU countries are already conducting national soil surveys to monitor changes in abiotic and biotic parameters, including endpoints related to soil functions. Nonetheless, the implementation of microbial endpoints in national soil surveys depends on the availability of standardized methods. However, despite progresses done (Thiele-Bruhn 2021) there is still a huge challenge to provide new standardized approaches, reference bioindicators and guidelines related to soil microorganisms (Djemiel et al. 2022). This observation is striking with regards to the EU water framework directive, the most significant European water legislation to date, which only considers diatom biodiversity as a microbial endpoint to assess the biological quality of water bodies, and toxicity tests on microalgae to establish environmental quality standards to chemicals, microbial functions or other microorganisms being totally disregarded (Pesce et al. 2020a).

### Microorganisms as nature-based solutions for pollution treatment

The scientific community faces several challenges to enable more widespread use of microorganisms for bioremediation of contaminated environmental matrices. Based on our fundamental understanding of the biodegradation or biotransformation of pollutants (see dedicated section), many recent examples demonstrate the effectiveness of using microorganisms for the bioremediation of soils and waters polluted by hydrocarbons or organohalides (McCarty et al. 2020, Naeem and Qazi 2020). However, there is still a long way to go to be able to propose bioremediation techniques for emerging or recalcitrant organic pollutants such as (micro-) plastics or PFAS, whose kinetics and degradation pathways are still understudied (Zhou et al. 2022). Although less commonly applied to metallic pollutants (particularly at large scale), bioremediation can effectively remove metals or metalloids from mine waste, soils, or waters through immobilization or transformation processes (Rahman and Singh 2020, Jacob et al. 2022, Nivetha et al. 2023). For example, bacterially mediated treatment of arsenic-rich acid mine waters has recently been successfully up-scaled from the lab to the field (Diaz-Vanegas et al. 2022). Moreover, phytoextraction of metals is more developed and can be efficiently improved through the action of microbes such as mycorrhizal fungi or plant growth promoting (PGP) bacteria that could enhance the speed and quantity of metal uptake by plants (Kazemalilou et al. 2020).

The different bioremediation approaches applied to soils or (ground)water are natural attenuation, bioaugmentation, biostimulation, and rhizostimulation (Khan et al. 2004). Natural attenuation, which requires that pollutants are being immobilized or degraded by natural processes (biotic or abiotic) without any human intervention, can be slow compared to bioaugmentation or biostimulation, and require long-term monitoring (Khan et al. 2004). Bioaugmentation consists of growing selected microorganisms with a known ability to degrade or transform a target pollutant. Although its effectiveness depends on the survival and development of inoculated strains, bioaugmentation can be effective, fast, and affordable as a ‘green’ clean-up option (Nwankwegu et al. 2022). A pitfall for bioaugmentation is the unforeseen interactions on added degrading strains with autochthonous microbes (Yu et al. 2005). Biostimulation aims to overcome factors limiting the activity of autochthonous microorganisms through the supply of nutrients (nitrogen source, electron acceptors/donors, and so on), surfactants, and/or oxygen. This requires a good knowledge of the indigenous communities and their physiological and metabolic needs. The optimal C/N/P ratio and bioavailability of pollutant must be determined and adjusted, and the stimulation of other populations that can out-compete the target microorganisms is not excluded (Adams et al. 2015).

In addition to the fact that bioremediation methods may be slower than more conventional physico-chemical approaches, microbial activity is also under the complex and tight influence of many environmental factors, and therefore, difficult to predict (Bala et al. 2022). To improve the reliability and the sustainability of bioremediation performance *in situ*, it is now crucial to better understand the factors driving microbial activity (Laroche et al. 2018, Diaz-Vanegas et al. 2022).

New bioremediation approaches have recently emerged such as microbial enzymes (Sharma et al. 2018, Saravanan et al. 2021), microbially assisted phytoremediation (Thijs et al. 2016, Sharma 2021, Yang et al. 2022), preventive bioremediation (Carles et al. 2021), encapsulation of microorganisms (Valdivia-Rivera et al. 2021), biosurfactants (Eras-Muñoz et al. 2022), and microbial nanotechnology/nanobioremediation (Mandeep and Shukla 2020, Hussain et al. 2022).

Another use of microorganisms that represents an innovative exploitation of microbe–metal interactions is the recovery of critical metals from secondary sources. This results in environmental clean-up and contributes to recycling. As cost is often an important obstacle for remediation, strategies integrating bioremediation and recovery of pollutants of economic interest such as metals hold great promise for the environmental and economic sectors (Guezennec et al. 2015, Bryan et al. 2020, Hubau et al. 2020, Gavrilescu 2022).

These previous examples highlight the persisting fundamental need to develop new isolation approaches (bacterial trapping, new culture media, and high throughput culturomics) in order to have a greater diversity of microbial strains degrading or transforming pollutants for bioaugmentation applications. One future challenge is to explore the higher potential of microbial consortia rather than individual strains and to be able to conserve these consortia and their properties on the long-term. In addition, improved methods are also needed to screen, characterize, produce, formulate, test, and validate degrading inoculants for cleaning up polluted soils (Duran et al. 2022). From a functional point of view, understanding the dynamics of microbial communities in these systems and how they can be stable and effective over time will be essential to engineer well-built and sustainable bioremediation systems. This in turn will help with the demands of the ecological transition to address identified planetary boundaries (Persson et al. 2022, Arp et al. 2023). The application of integrative approaches could be useful in this respect, as shown in a recent study by Hellal et al. (2021) on the monitoring of *in situ* natural attenuation of a multipolluted aquifer. Enrichment, isolation and preservation of efficient microbial strains capable of degrading various organic pollutants is still necessary. The creation of an open repository of adequately characterized degrading strains could facilitate the choice of the most effective isolates depending on the pollutant to be biodegraded and the physico-chemical conditions of the environment to be remediated. A few recent initiatives are working towards this goal (e.g. the EU Horizon project MIBIREM), in particular on the preservation of microbial consortia whose preservation, stability, and maintenance of activity over time remains a challenge.

## Concluding remarks

The anthropocene is characterized by global chemical pollution as underlined by the International Panel on Chemical Pollution (IPCP). Microbial communities, through their responses to exposure to pollutants and their biotransformation capabilities, represent sensitive bioindicators for revealing the ecological quality of the environment and are promising actors for the remediation of polluted environments. Consequently, microbial ecotoxicology has become a key(stone) area for scientific research as it fills the knowledge gaps necessary to implement a strategy taking on board microbial communities in order to monitor and implement the ‘One Health’ agenda. One of the challenges for microbial ecotoxicologists is now to embed their work on the fate and effects of pollutants in a perspective that simultaneously embraces ecosystem taxonomy and functions, ecosystem services, and nature-based solutions (Peixoto et al. 2022, Lemke and DeSalle 2023), and which integrates multistress situations related to global changes (Sabater et al. 2019). In addition to these new knowledge inputs and contributions, microbial ecotoxicologists will also assist in the definition of NOR and threshold values of acceptable effects of pollutants. This knowledge can then be integrated and proposed to stakeholders for implementation in new regulations more protective of One Health. To achieve this objective, microbial ecotoxicologists will represent a driving force to propose innovative concepts, approaches and (standardized) methods, open science data sets and scientific expertise that can be further mobilized by socio-economic partners. To increase their visibility and their impact, microbial ecotoxicologists will have a key role in further promoting and developing interdisciplinarity. Furthermore, they will have the responsibility of training a new generation of scientists aware of the importance of microbial communities within the ‘One Health’ framework and capable of scientific mediation towards diverse players of the society, in particular stakeholders, politicians, and elected representatives who have in their hands the power to implement new regulations and impulse new directions in favour of a more sustainable world.

Taken together, the methodological challenges identified in order to adequately assess the biological effects of chemical pollution will require more and improved integrative studies. These will cover a larger diversity of microbial groups, more directly link the microbiome to its function, and combine novel and/or traditional methods with statistical and modelling approaches. All these tools hold strong promise for the field of microbial ecotoxicology, as they will allow the characterization of the effects and fate of toxic chemicals at the ecosystem scale as well as the taxonomic, functional, and morphometric responses of microbial communities. In turn, this should also allow easier consideration of space and time in environmental studies in the future, through long-term monitoring and original experimental designs considering the complexity of real-world environments.

## References

[bib2] Achermann S , Mansfeldt CB, Müller M et al. Relating metatranscriptomic profiles to the micropollutant biotransformation potential of complex microbial communities. Environ Sci Technol. 2020;54:235–44.. 10.1021/acs.est.9b05421. 31774283

[bib3] Adamovsky O , Buerger AN, Wormington AM et al. The gut microbiome and aquatic toxicology: an emerging concept for environmental health. Environ Toxicol Chem. 2018;37:2758–75.. 10.1002/etc.4249. 30094867

[bib4] Adams GO , Fufeyin PT, Okoro SE et al. Bioremediation, biostimulation and bioaugmention: a review. Int J Environ Bioremediat Biodegrad. 2015;3:28–39.. 10.12691/ijebb-3-1-5.

[bib5] Adrian L , Löffler FE. Organohalide-respiring bacteria—an introduction. In: Adrian L, Löffler FE, (eds), Organohalide-Respiring Bacteria. Berlin, Heidelberg: Springer, 2016, 3–6.. 10.1007/978-3-662-49875-0_1.

[bib6] Ahemad M , Khan MS. Comparative toxicity of selected insecticides to pea plants and growth promotion in response to insecticide-tolerant and plant growth promoting *Rhizobium leguminosarum*. Crop Prot. 2010;29:325–9.. 10.1016/j.cropro.2010.01.005.

[bib7] Allison SD , Martiny JBH. Resistance, resilience, and redundancy in microbial communities. Proc Natl Acad Sci. 2008;105:11512–9.. 10.1073/pnas.0801925105. 18695234 PMC2556421

[bib8] Almeida E , FC M, Lage OM. Culturomics remains a highly valuable methodology to obtain rare microbial diversity with putative biotechnological potential from two Portuguese salterns. Front Biosci Elite. 2022;14:11. 10.31083/j.fbe1402011. 35730452

[bib9] Álvarez-Barragán J , Cravo-Laureau C, Duran R. Fungal-bacterial network in PAH–contaminated coastal marine sediment. Environ Sci Pollut Res. 2022;29:72718–28.. 10.1007/s11356-022-21012-4. 35614354

[bib10] Argudo M , Gich F, Bonet B et al. Responses of resident (DNA) and active (RNA) microbial communities in fluvial biofilms under different polluted scenarios. Chemosphere. 2020;242:125108. 10.1016/j.chemosphere.2019.125108 31669992

[bib11] Arp HPH , Aurich D, Schymanski EL et al. Avoiding the next silent spring: our chemical past, present, and future. Environ Sci Technol. 2023;57. 10.1021/acs.est.3c01735. PMC10134483 37053515

[bib1] Aßhauer KP , Wemheuer B, Daniel R et al. Tax4Fun: predicting functional profiles from metagenomic 16S rRNA data. Bioinformatics. 2015;31:2882–4.. 10.1093/bioinformatics/btv287. 25957349 PMC4547618

[bib12] Bala S , Garg D, Thirumalesh BV et al. Recent strategies for bioremediation of emerging pollutants: a review for a green and sustainable environment. Toxics. 2022;10:484. 10.3390/toxics10080484. 36006163 PMC9413587

[bib13] Barkay T , Miller S, Summers A. Bacterial mercury resistance from atoms to ecosystems. FEMS Microbiol Rev. 2003;27:355–84.. 10.1016/S0168-6445(03)00046-9. 12829275

[bib14] Barra Caracciolo A , Terenzi V. Rhizosphere microbial communities and heavy metals. Microorganisms. 2021;9:1462. 10.3390/microorganisms9071462. 34361898 PMC8307176

[bib15] Barral-Fraga L , Barral MT, MacNeill KL et al. Biotic and abiotic factors influencing arsenic biogeochemistry and toxicity in fluvial ecosystems: a review. Int J Environ Res Public Health. 2020;17:2331. 10.3390/ijerph17072331. 32235625 PMC7177459

[bib26] Bérard A , Capowiez L, Mombo S et al. Soil microbial respiration and PICT responses to an industrial and historic lead pollution: a field study. Environ Sci Pollut Res. 2016;23:4271–81.. 10.1007/s11356-015-5089-z. 26233741

[bib16] Berg G , Rybakova D, Fischer D et al. Microbiome definition re-visited: old concepts and new challenges. Microbiome. 2020;8:103. 10.1186/s40168-020-00875-0. 32605663 PMC7329523

[bib18] Birrer SC , Dafforn KA, Johnston EL. Microbial community responses to contaminants and the use of molecular techniques. In: Cravo-Laureau C, Cagnon C, Lauga B, Duran R, (eds), Microbial Ecotoxicology. Cham: Springer International Publishing, 2017, 165–83.. 10.1007/978-3-319-61795-4_8.

[bib17] Blanck H , Wangberg SA, Molander S. Pollution-induced community tolerance – a new ecotoxicological tool. functional testing of aquatic biota for estimating hazards of chemicals. Am Soc Test Mater. 1988;988:219–23.

[bib19] Bonnineau C , Artigas J, Chaumet B et al. Role of biofilms in contaminant bioaccumulation and trophic transfer in aquatic ecosystems: current state of knowledge and future challenges. In: de Voogt P, (ed.), Reviews of Environmental Contamination and Toxicology. Vol. 253. Cham: Springer International Publishing, 2021, 115–53.. 10.1007/398_2019_39. 32166435

[bib20] Borchert E , Hammerschmidt K, Hentschel U et al. Enhancing microbial pollutant degradation by integrating eco-evolutionary principles with environmental biotechnology. Trends Microbiol. 2021;29:908–18.. 10.1016/j.tim.2021.03.002. 33812769

[bib21] Bouchez T , Blieux AL, Dequiedt S et al. Molecular microbiology methods for environmental diagnosis. Environ Chem Lett. 2016;14:423–41.. 10.1007/s10311-016-0581-3.

[bib22] Bourhane Z , Lanzén A, Cagnon C et al. Microbial diversity alteration reveals biomarkers of contamination in soil-river-lake continuum. J Hazard Mater. 2022;421:126789. 10.1016/j.jhazmat.2021.126789. 34365235

[bib23] Brock T , Bigler F, Frampton G et al. Ecological recovery and resilience in environmental risk assessments at the European Food Safety Authority. Integr Environ Assess Manag. 2018;14:586–91.. 10.1002/ieam.4079. 30489025

[bib24] Bryan CG , Williamson BJ, Całus-Moszko J et al. CEReS – co-processing of coal mine & electronic wastes: novel resources for a sustainable future. Hydrometallurgy. 2020;197:105444. 10.1016/j.hydromet.2020.105444.

[bib25] Bungau S , Behl T, Aleya L et al. Expatiating the impact of anthropogenic aspects and climatic factors on long-term soil monitoring and management. Environ Sci Pollut Res. 2021;28:30528–50.. 10.1007/s11356-021-14127-7. 33905061

[bib27] Campillo-Cora C , Soto-Gómez D, Arias-Estévez M et al. Bacterial community tolerance to Cu in soils with geochemical baseline concentrations (GBCs) of heavy metals: importance for pollution induced community tolerance (PICT) determinations using the leucine incorporation method. Soil Biol Biochem. 2021;155:108157. 10.1016/j.soilbio.2021.108157.

[bib28] Carles L , Martin-Laurent F, Devers M et al. Potential of preventive bioremediation to reduce environmental contamination by pesticides in an agricultural context: a case study with the herbicide 2,4-D. J Hazard Mater. 2021;416:125740. 10.1016/j.jhazmat.2021.125740. 33848793

[bib42] Cébron A , Zeghal E, Usseglio-Polatera P et al. BactoTraits – a functional trait database to evaluate how natural and man-induced changes influence the assembly of bacterial communities. Ecol Indic. 2021;130:108047. 10.1016/j.ecolind.2021.108047.

[bib31] Châtillon E , Duran R, Rigal F et al. New insights into microbial community coalescence in the land-sea continuum. Microbiol Res, 2023;267:127259. 10.1016/j.micres.2022.127259. 36436444

[bib29] Chaudhary DK , Khulan A, Kim J. Development of a novel cultivation technique for uncultured soil bacteria. Sci Rep. 2019;9, 6666. 10.1038/s41598-019-43182-x. 31040339 PMC6491550

[bib30] Chishti Z , Ahmad Z, Zhang X et al. Optimization of biotic and abiotic factors liable for biodegradation of chlorpyrifos and their modeling using neural network approaches. Appl Soil Ecol. 2021;166:103990. 10.1016/j.apsoil.2021.103990.

[bib32] Claus SP , Guillou H, Ellero-Simatos S. The gut microbiota: a major player in the toxicity of environmental pollutants?. npj Biofilms Microbiomes. 2016;2, 16003. 10.1038/npjbiofilms.2016.3. 28721242 PMC5515271

[bib33] Codello A , Hose GC, Chariton A. Microbial co-occurrence networks as a biomonitoring tool for aquatic environments: a review. Mar Freshw Res. 2023;74:409–22.. 10.1071/MF22045.

[bib34] Cordier T , Frontalini F, Cermakova K et al. Multi-marker eDNA metabarcoding survey to assess the environmental impact of three offshore gas platforms in the North Adriatic Sea (Italy). Mar Environ Res. 2019;146:24–34. 30890270 10.1016/j.marenvres.2018.12.009

[bib35] Costa S , Lopes I, Proença DN et al. Diversity of cutaneous microbiome of *Pelophylax perezi* populations inhabiting different environments. Sci Total Environ. 2016;572:995–1004.. 10.1016/j.scitotenv.2016.07.230. 27522290

[bib36] Courcoul C , Leflaive J, Ferriol J et al. The sensitivity of aquatic microbial communities to a complex agricultural contaminant depends on previous drought conditions. Water Res. 2022;217:118396. 10.1016/j.watres.2022.118396. 35413563

[bib37] Crampon M , Hellal J, Mouvet C et al. Do natural biofilm impact nZVI mobility and interactions with porous media? A column study. Sci Total Environ. 2018;610-611:709–19.. 10.1016/j.scitotenv.2017.08.106. 28822938

[bib38] Cravo-Laureau C , Duran R. Marine coastal sediments microbial hydrocarbon degradation processes: contribution of experimental ecology in the omics' era. Front Microbiol. 2014;5. 10.3389/fmicb.2014.00039. PMC3921567 24575083

[bib39] Cravo-Laureau C , Lauga B, Cagnon C et al. Microbial responses to pollution—ecotoxicology: introducing the different biological levels. In: Cravo-Laureau C, Cagnon C, Lauga B, Duran R, (eds), Microbial Ecotoxicology. Cham: Springer International Publishing, 2017, 45–62.. 10.1007/978-3-319-61795-4_4.

[bib40] Crouzet O , Consentino L, Pétraud J-P et al. Soil photosynthetic microbial communities mediate aggregate stability: influence of cropping systems and herbicide use in an agricultural soil. Front Microbiol. 2019;10. 10.3389/fmicb.2019.01319. PMC6587365 31258520

[bib41] Cuevas DA , Edirisinghe J, Henry CS et al. From DNA to FBA: how to build your own genome-scale metabolic model. Front Microbiol. 2016;7. 10.3389/fmicb.2016.00907. PMC4911401 27379044

[bib56] Dávila D , Esquivel JP, Sabaté N et al. Silicon-based microfabricated microbial fuel cell toxicity sensor. Biosens Bioelectron. 2011;26:2426–30.. 10.1016/j.bios.2010.10.025. 21074397

[bib43] Delgado-Baquerizo M , Maestre FT, Reich PB et al. Microbial diversity drives multifunctionality in terrestrial ecosystems. Nat Commun. 2016;7:art. 10541. 10.1038/ncomms10541. PMC4738359 26817514

[bib44] Delgado-Baquerizo M , Reich PB, Trivedi C et al. Multiple elements of soil biodiversity drive ecosystem functions across biomes. Nat Ecol Evol. 2020;4:210–20.. 10.1038/s41559-019-1084-y. 32015427

[bib45] Deng R , Chen Y, Deng X et al. A critical review of resistance and oxidation mechanisms of Sb-oxidizing bacteria for the bioremediation of Sb(III) pollution. Front Microbiol. 2021;12. 10.3389/fmicb.2021.738596. PMC8453088 34557178

[bib46] Diaz-Vanegas C , Casiot C, Lin L et al. Performance of semi-passive systems for the biological treatment of high-as acid mine drainage: results from a year of monitoring at the Carnoulès Mine (Southern France). Mine Water Environ. 2022;41:679–94.. 10.1007/s10230-022-00885-4.

[bib47] Djemiel C , Dequiedt S, Karimi B et al. Potential of meta-omics to provide modern microbial indicators for monitoring soil quality and securing food production. Front Microbiol. 2022;13. 10.3389/fmicb.2022.889788. PMC9280627 35847063

[bib48] Dolinová I , Czinnerová M, Dvořák L et al. Dynamics of organohalide-respiring bacteria and their genes following in-situ chemical oxidation of chlorinated ethenes and biostimulation. Chemosphere. 2016;157:276–85.. 10.1016/j.chemosphere.2016.05.030. 27236848

[bib49] Dong H , Huang L, Zhao L et al. A critical review of mineral–microbe interaction and co-evolution: mechanisms and applications. Natl Sci Rev. 2022;9:nwac128. 10.1093/nsr/nwac128. 36196117 PMC9522408

[bib50] Douglas GM , Maffei VJ, Zaneveld JR et al. PICRUSt2 for prediction of metagenome functions. Nat Biotechnol. 2020;38:685–8.. 10.1038/s41587-020-0548-6 32483366 PMC7365738

[bib51] Douglas GML , Morgan GI. A primer and discussion on DNA-based microbiome data and related bioinformatics analyses. Peer Comm J. 2021;1. 10.24072/pcjournal.2.

[bib52] Duperron S , Halary S, Gallet A et al. Microbiome-aware ecotoxicology of organisms: relevance, pitfalls, and challenges. Front Pub Health. 2020;8. 10.3389/fpubh.2020.00407. PMC7472533 32974256

[bib53] Duran C , Zhang S, Yang C et al. Low-cost gel-filled microwell array device for screening marine microbial consortium. Front Microbiol. 2022;13. 10.3389/fmicb.2022.1031439. PMC9800614 36590440

[bib54] Duran R , Cravo-Laureau C. Role of environmental factors and microorganisms in determining the fate of polycyclic aromatic hydrocarbons in the marine environment. FEMS Microbiol Rev. 2016;40:814–30.. 10.1093/femsre/fuw031. 28201512 PMC5091036

[bib55] Durand MJ , Hua A, Jouanneau S et al. Detection of metal and organometallic compounds with bioluminescent bacterial bioassays. In: Thouand G, Marks R, (eds), Bioluminescence: Fundamentals and Applications in Biotechnology. Vol. 3. Cham: Springer International Publishing, 2016, 77–99.. 10.1007/10_2015_332.

[bib57] Egal M , Casiot C, Morin G et al. An updated insight into the natural attenuation of As concentrations in Reigous Creek (southern France). Appl Geochem. 2010;25:1949–57.. 10.1016/j.apgeochem.2010.10.012.

[bib58] Eras-Muñoz E , Farré A, Sánchez A et al. Microbial biosurfactants: a review of recent environmental applications. Bioengineered. 2022;13:12365–91.. 10.1080/21655979.2022.2074621. 35674010 PMC9275870

[bib59] Escher BI , Altenburger R, Blüher M et al. Modernizing persistence–bioaccumulation–toxicity (PBT) assessment with high throughput animal-free methods. Arch Toxicol. 2023;97:1267–83.. 10.1007/s00204-023-03485-5. 36952002 PMC10110678

[bib61] European Food Safety A . Dietary reference values for nutrients summary report. EFSA Support Publ. 2017;14:e15121E. 10.2903/sp.efsa.2017.e15121.

[bib60] European Food Safety A . The 2010 European Union Report on pesticide residues in food. EFSA J. 2013;11:3130. 10.2903/j.efsa.2013.3130. PMC11883121 40061627

[bib62] Eymard-Vernain E , Lelong C et al. Impact of a model soil microorganism and of its secretome on the fate of silver nanoparticles. Environ Sci Technol. 2018;52:71–8.. 10.1021/acs.est.7b04071. 29211460

[bib63] Fan Y , Chen J, Shirkey G et al. Applications of structural equation modeling (SEM) in ecological studies: an updated review. Ecol Process. 2016;5:art. 19. 10.1186/s13717-016-0063-3.

[bib64] Fang D , Gao G, Yang Y et al. Redox mediator-based microbial biosensors for acute water toxicity assessment: a critical review. Chem Electro Chem. 2020;7:2513–26.. 10.1002/celc.202000367.

[bib65] Fei Y , Huang S, Zhang H et al. Response of soil enzyme activities and bacterial communities to the accumulation of microplastics in an acid cropped soil. Sci Total Environ. 2020;707:135634. 10.1016/j.scitotenv.2019.135634. 31761364

[bib66] Francioli D , Lentendu G, Lewin S et al. DNA metabarcoding for the characterization of terrestrial microbiota-pitfalls and solutions. Microorganisms. 2021;9:art. 361. 10.3390/microorganisms9020361. 33673098 PMC7918050

[bib67] Gadd GM . Metals, minerals and microbes: geomicrobiology and bioremediation. Microbiology. 2010;156:609–43.. 10.1099/mic.0.037143-0. 20019082

[bib68] Gallois N , Dhommée R, Braylé P et al. Federating young researchers in microbial ecotoxicology: ecotoxicoMicYR 2021, the first international webinar organized for and by young microbial ecotoxicology researchers. Environ Sci Pollut Res. 2022;29:65880–5.. 10.1007/s11356-022-22410-4. 35972659

[bib69] Garland G , Banerjee S, Edlinger A et al. A closer look at the functions behind ecosystem multifunctionality: a review. J Ecol. 2021;109:600–13.. 10.1111/1365-2745.13511.

[bib70] Gavrilescu M . Microbial recovery of critical metals from secondary sources. Bioresour Technol. 2022;344:126208. 10.1016/j.biortech.2021.126208. 34715340

[bib71] Gerbersdorf SU , Jancke T, Westrich B. Physico-chemical and biological sediment properties determining erosion resistance of contaminated riverine sediments – temporal and vertical pattern at the Lauffen reservoir/River Neckar, Germany. Limnologica. 2005;35:132–44.. 10.1016/j.limno.2005.05.001

[bib72] Ghiglione J-F , Martin-Laurent F, Pesce S. Microbial ecotoxicology: an emerging discipline facing contemporary environmental threats. Environ Sci Pollut Res. 2016;23:3981–3.. 10.1007/s11356-015-5763-1. 26578376

[bib73] Ghiglione J-F , Martin-Laurent F, Stachowski-Haberkorn S et al. The coming of age of microbial ecotoxicology: report on the first two meetings in France. Environ Sci Pollut Res. 2014;21:14241–5.. 10.1007/s11356-014-3390-x. 25096491

[bib74] Graham EB , Knelman JE, Schindlbacher A et al. Microbes as engines of ecosystem function: when does community structure enhance predictions of ecosystem processes?. Front Microbiol. 2016;7. 10.3389/fmicb.2016.00214. PMC4764795 26941732

[bib75] Greenspan SE , Peloso P, Fuentes-González JA et al. Low microbiome diversity in threatened amphibians from two biodiversity hotspots. Anim Microbiome. 2022;4:69. 10.1186/s42523-022-00220-w. 36582011 PMC9801548

[bib76] Guasch H , Bernal S, Bruno D et al. Interactions between microplastics and benthic biofilms in fluvial ecosystems: knowledge gaps and future trends. Freshwat Sci. 2022;41:442–58.. 10.1086/721472.

[bib77] Guezennec A-G , Bru K, Jacob J et al. Co-processing of sulfidic mining wastes and metal-rich post-consumer wastes by biohydrometallurgy. Miner Eng. 2015;75:45–53.. 10.1016/j.mineng.2014.12.033.

[bib78] Gutiérrez L , Garbisu C, Ciprián E et al. Application of ecological risk assessment based on a novel TRIAD-tiered approach to contaminated soil surrounding a closed non-sealed landfill. Sci Total Environ. 2015;514:49–59.. 10.1016/j.scitotenv.2015.01.103. 25659305

[bib79] Han Y , Zhang J, Hu CQ et al. In silico ADME and toxicity prediction of ceftazidime and its impurities. Front Pharmacol. 2019;10:434. 10.3389/fphar.2019.00434. 31068821 PMC6491819

[bib80] Hatzenpichler R , Krukenberg V, Spietz RL et al. Next-generation physiology approaches to study microbiome function at single cell level. Nat Rev Microbiol. 2020;18:241–56.. 10.1038/s41579-020-0323-1. 32055027 PMC7133793

[bib81] Hayes F , Spurgeon DJ, Lofts S et al. Evidence-based logic chains demonstrate multiple impacts of trace metals on ecosystem services. J Environ Manage. 2018;223:150–164.. 10.1016/j.jenvman.2018.05.053. 29929071

[bib82] He Z , Deng Y, Van Nostrand JD et al. GeoChip 3.0 as a high-throughput tool for analyzing microbial community composition, structure and functional activity. ISME J. 2010;4:1167–79.. 10.1038/ismej.2010.46. 20428223

[bib83] He Z , Van Nostrand JD, Zhou J. Applications of functional gene microarrays for profiling microbial communities. Curr Opin Biotechnol. 2012;23:460–6.. 10.1016/j.copbio.2011.12.021. 22226464

[bib84] Hellal J , Guédron S, Huguet L et al. Mercury mobilization and speciation linked to bacterial iron oxide and sulfate reduction: a column study to mimic reactive transfer in an anoxic aquifer. J Contam Hydrol. 2015;180:56–68.. 10.1016/j.jconhyd.2015.08.001. 26275395

[bib85] Hellal J , Joulian C, Urien C et al. Chlorinated ethene biodegradation and associated bacterial taxa in multi-polluted groundwater: Insights from biomolecular markers and stable isotope analysis. Sci Total Environ. 2021;763:142950. 10.1016/j.scitotenv.2020.142950. 33127155

[bib86] Henry CS , Bernstein HC, Weisenhorn P et al. Microbial community metabolic modeling: a community data-driven network reconstruction. J Cell Physiol. 2016;231:2339–45.. 10.1002/jcp.25428. 27186840 PMC5132105

[bib87] Hernández Medina R , KutuzovaS, NielsenKNet al. Machine learning and deep learning applications in microbiome research. ISME Commun. 2022;2:98. 10.1038/s43705-022-00182-9.37938690

[bib88] Herold M , Martínez Arbas SM, Narayanasamy S et al. Integration of time-series meta-omics data reveals how microbial ecosystems respond to disturbance. Nat Commun. 2020;11:art. 5281. 10.1038/s41467-020-19006-2. PMC7572474 33077707

[bib92] Héry M , Casiot C, Resongles E et al. Release of arsenite, arsenate and methyl-arsenic species from streambed sediment affected by acid mine drainage: a microcosm study. Environ Chem. 2014;11:514–24.. 10.1071/EN13225.

[bib93] Héry M , Rizoulis A, Sanguin H et al. Microbial ecology of arsenic-mobilizing Cambodian sediments: lithological controls uncovered by stable-isotope probing. Environ Microbiol. 2015;17:1857–69.. 10.1111/1462-2920.12412. 24467551

[bib89] Hidalgo KJ , Sierra-Garcia IN, Dellagnezze BM et al. Metagenomic insights into the mechanisms for biodegradation of polycyclic aromatic hydrocarbons in the oil supply chain. Front Microbiol. 2020;11. 10.3389/fmicb.2020.561506. PMC7530279 33072021

[bib94] Hollender J , Schymanski EL, Singer HP et al. Nontarget screening with high resolution mass spectrometry in the environment: ready to go?. Environ Sci Technol. 2017;51:11505–12.. 10.1021/acs.est.7b02184. 28877430

[bib90] Hubau A , Guezennec A-G, Joulian C et al. Bioleaching to reprocess sulfidic polymetallic primary mining residues: determination of metal leaching mechanisms. Hydrometallurgy. 2020;197:105484. 10.1016/j.hydromet.2020.105484.

[bib91] Hussain A , Rehman F, Rafeeq H et al. In-situ, ex-situ, and nano-remediation strategies to treat polluted soil, water, and air – a review. Chemosphere. 2022;289:133252. 10.1016/j.chemosphere.2021.133252. 34902385

[bib95] Jacob J , Joulian C, Battaglia-Brunet F. Start-up and performance of a full scale passive system in-cluding biofilters for the treatment of Fe, as and Mn in a neutral mine drainage. Water. 2022;14:1963. 10.3390/w14121963.

[bib96] Jessup CM , Forde SE, Bohannan BJM. Microbial experimental systems in ecology. advances in ecological research. Acad Press. 2005;37:273–307.. 10.1016/S0065-2504(04)37009-1.

[bib97] Jessup CM , Kassen R, Forde SE et al. Big questions, small worlds: microbial model systems in ecology. Trends Ecol Evol. 2004;19:189–97.. 10.1016/j.tree.2004.01.008. 16701253

[bib98] Jia K , Eltzov E, Toury T et al. A lower limit of detection for atrazine was obtained using bioluminescent reporter bacteria via a lower incubation temperature. Ecotoxicol Environ Saf. 2012;84:221–6.. 10.1016/j.ecoenv.2012.07.009. 22858105

[bib99] Jiang Y , Yang X, Liang P et al. Microbial fuel cell sensors for water quality early warning systems: fundamentals, signal resolution, optimization and future challenges. Renew Sustain Energy Rev. 2018;81:292–305.. 10.1016/j.rser.2017.06.099.

[bib100] Kakumanu ML , Reeves AM, Anderson TD et al. Honey bee gut microbiome is altered by in-hive pesticide exposures. Front Microbiol. 2016;7. 10.3389/fmicb.2016.01255. PMC4985556 27579024

[bib101] Karpouzas DG , Papadopoulou E, Ipsilantis I et al. Effects of nicosulfuron on the abundance and diversity of arbuscular mycorrhizal fungi used as indicators of pesticide soil microbial toxicity. Ecol Indic. 2014;39:44–53.. 10.1016/j.ecolind.2013.12.004.

[bib102] Kazemalilou S , Delangiz N, Asgari Lajayer B et al. Chapter 9 - insight into plant-bacteria-fungi interactions to improve plant performance via remediation of heavy metals: an overview. In: Sharma V, Salwan R, Al-Ani LKT, (eds), Molecular Aspects of Plant Beneficial Microbes in Agriculture. Cambridge: Academic Press, 2020, 123–32.. 10.1016/B978-0-12-818469-1.00010-9.

[bib103] Khan FI , Husain T, Hejazi R. An overview and analysis of site remediation technologies. J Environ Manage. 2004;71:95–122.. 10.1016/j.jenvman.2004.02.003. 15135946

[bib104] Kimbell LK , LaMartina EL, Kappell AD et al. Cast iron drinking water pipe biofilms support diverse microbial communities containing antibiotic resistance genes, metal resistance genes, and class 1 integrons. Environ Sci. 2021;7:584–98.. 10.1039/D0EW01059F.

[bib105] Klimkowicz-Pawlas A , Maliszewska-Kordybach B, Smreczak B. Triad-based screening risk assessment of the agricultural area exposed to the long-term PAHs contamination. Environ Geochem Health. 2019;41:1369–85.. 10.1007/s10653-018-0220-y. 30467649 PMC6702193

[bib106] Kloster M , Langenkämper D, Zurowietz M et al. Deep learning-based diatom taxonomy on virtual slides. Sci Rep. 2020;10:14416. 10.1038/s41598-020-71165-w. 32879374 PMC7468105

[bib107] Laforest-Lapointe I , Paquette A, Messier C et al. Leaf bacterial diversity mediates plant diversity and ecosystem function relationships. Nature. 2017;546:145–7.. 10.1038/nature22399. 28538736

[bib108] Lapanje A , Zrimec A, Drobne D et al. Long-term Hg pollution-induced structural shifts of bacterial community in the terrestrial isopod (*Porcellio scaber*) gut. Environ Pollut. 2010;158:3186–93.. 10.1016/j.envpol.2010.07.001. 20724045

[bib109] Laroche E , Casiot C, Fernandez-Rojo L et al. Dynamics of bacterial communities mediating the treatment of an As-rich acid mine drainage in a field pilot. Front Microbiol. 2018;9:3169. 10.3389/fmicb.2018.03169. 30627121 PMC6309452

[bib110] Lavoie I , Morin S, Laderriere V et al. Freshwater diatoms as indicators of combined long-term mining and urban stressors in Junction Creek (Ontario, Canada). Environments. 2018;5. 10.3390/environments5020030.

[bib111] Lecerf A , Cébron A, Gilbert F et al. Using plant litter decomposition as an indicator of ecosystem response to soil contamination. Ecol Indic. 2021;125:107554. 10.1016/j.ecolind.2021.107554.

[bib112] Lemke M , DeSalle R. The next generation of microbial ecology and its importance in environmental sustainability. Microb Ecol. 2023; 85. 10.1007/s00248-023-02185-y. PMC10156817 36826587

[bib113] Lemmel F , Maunoury-Danger F, Fanesi A et al. Soil properties and multi-pollution affect taxonomic and functional bacterial diversity in a range of french soils displaying an anthropisation gradient. Microb Ecol. 2019a;77:993–1013.. 10.1007/s00248-018-1297-7. 30467715

[bib115] Lemmel F , Maunoury-Danger F, Leyval C et al. Altered fungal communities in contaminated soils from French industrial brownfields. J Hazard Mater. 2021;406:124296. 10.1016/j.jhazmat.2020.124296. 33268205

[bib114] Lemmel F , Maunoury-Danger F, Leyval C et al. DNA stable isotope probing reveals contrasted activity and phenanthrene-degrading bacteria identity in a gradient of anthropized soils. FEMS Microbiol Ecol. 2019b;95:fiz181. 10.1093/femsec/fiz181. 31730156

[bib116] Li H , Yao J, Min N et al. Relationships between microbial activity, enzyme activities and metal(loid) form in NiCu tailings area. Sci Total Environ. 2022;812:152326. 10.1016/j.scitotenv.2021.152326. 34906578

[bib117] Li X , Liu B, Zheng G et al. Deep-learning-based information mining from ocean remote-sensing imagery. Natl Sci. 2020;7:1584–605.. 10.1093/nsr/nwaa047. PMC8288802 34691490

[bib118] Li Y , Liang Y, Zhang H et al. Variation, distribution, and diversity of canonical ammonia-oxidizing microorganisms and complete-nitrifying bacteria in highly contaminated ecological restoration regions in the Siding mine area. Ecotoxicol Environ Saf. 2021;217:112274. 10.1016/j.ecoenv.2021.112274. 33930771

[bib119] Liu G , Dave PH, Kwong RWM et al. Influence of microplastics on the mobility, bioavailability, and toxicity of heavy metals: a review. Bull Environ Contam Toxicol. 2021;107:710–21.. 10.1007/s00128-021-03339-9. 34331555

[bib120] Loreau M , Naeem S, Inchausti P et al. Biodiversity and ecosystem functioning: current knowledge and future challenges. Science. 2001;294:804–8.. 10.1126/science.1064088. 11679658

[bib121] Lu L , Chen C, Ke T et al. Long-term metal pollution shifts microbial functional profiles of nitrification and denitrification in agricultural soils. Sci Total Environ. 2022;830:154732. 10.1016/j.scitotenv.2022.154732. 35346706

[bib122] Luo J , Guo X, Liang J et al. The influence of elevated CO_2_ on bacterial community structure and its co-occurrence network in soils polluted with Cr_2_O_3_ nanoparticles. Sci Total Environ. 2021;779:146430. 10.1016/j.scitotenv.2021.146430. 33752002

[bib123] Lyautey E , Bonnineau C, Billard P et al. Diversity, functions and antibiotic resistance of sediment microbial communities from Lake Geneva are driven by the spatial distribution of anthropogenic contamination. Front Microbiol. 2021;12:art. 738629. 10.3389/fmicb.2021.738629. PMC8560053 34733255

[bib133] McCarty PL , Criddle CS, Vogel TM. Retrospective on microbial transformations of halogenated organics. Environ Sci Process Impacts. 2020;22:512–7.. 10.1039/C9EM00575G. 32181779

[bib124] Madeira CL , de Araújo JC. Inhibition of anammox activity by municipal and industrial wastewater pollutants: a review. Sci Total Environ. 2021;799:149449. 10.1016/j.scitotenv.2021.149449. 34371406

[bib125] Mahamoud AA , Lyautey E, Bonnineau C et al. Environmental concentrations of copper, alone or in mixture with arsenic, can impact river sediment microbial community structure and functions. Front Microbiol. 2018;9:1852. 10.3389/fmicb.2018.01852. 30158909 PMC6104476

[bib126] Maitland VC , Robinson CV, Porter TM et al. Freshwater diatom biomonitoring through benthic kick-net metabarcoding. PLoS ONE. 2020;15:e0242143. 10.1371/journal.pone.0242143. 33206700 PMC7673570

[bib127] Malla MA , Dubey A, Yadav S et al. Understanding and designing the strategies for the microbe-mediated remediation of environmental contaminants using Omics approaches. Front Microbiol. 2018;9:2868. 10.3389/fmicb.2018.01132. 29915565 PMC5994547

[bib129] Marathe NP , Bank MS. The microplastic-antibiotic resistance connection. In: Bank MS (ed.), Microplastic in the Environment: Pattern and Process. Environmental Contamination Remediation and Management. Cham: Springer, 2022, 311–22.. 10.1007/978-3-030-78627-4.

[bib132] Martínez Arbas S , Busi SB, Queirós P et al. Challenges, strategies, and perspectives for reference-independent longitudinal multi-omic microbiome studies. Front Genet. 2021;12:666244. 10.3389/fgene.2021.666244. 34194470 PMC8236828

[bib130] Martini S , Larras F, Boyé A et al. Functional trait-based approaches as a common framework for aquatic ecologists. Limnol Oceanogr. 2021;66:965–94.. 10.1002/lno.11655.

[bib131] Martiny AC . High proportions of bacteria are culturable across major biomes. ISME J. 2019;13:2125–8.. 10.1038/s41396-019-0410-3. 30952994 PMC6775996

[bib134] Meena RS , Kumar S, Datta R et al. Impact of agrochemicals on soil microbiota and management: a review. Land. 2020;9:34. 10.3390/land9020034.

[bib135] Meziti A , Tsementzi D, Ar. Kormas K et al. Anthropogenic effects on bacterial diversity and function along a river-to-estuary gradient in Northwest Greece revealed by metagenomics. Environ Microbiol. 2016;18:4640–52.. 10.1111/1462-2920.13303. 27001690

[bib136] Mony C , Uroy L, Khalfallah F et al. Landscape connectivity for the invisibles. Ecography. 2022;2022:e06041. 10.1111/ecog.06041.

[bib138] Morin S , Artigas J. Twenty years of research in ecosystem functions in aquatic microbial ecotoxicology. Environ Toxicol Chem. 2023;42:1867–88.. 10.1002/etc.57081 37401851

[bib137] Morin S , Bottin M, Mazzella N et al. Linking diatom community structure to pesticide input as evaluated through a spatial contamination potential (Phytopixal): a case study in the Neste river system (South-West France). Aquat Toxicol. 2009;94:28–39.. 10.1016/j.aquatox.2009.05.012. 19535156

[bib139] Morris A , Meyer K, Bohannan B. Linking microbial communities to ecosystem functions: what we can learn from genotype–phenotype mapping in organisms. Philos Trans R Soc B Biol Sci. 2020;375:20190244. 10.1098/rstb.2019.0244. PMC7133535 32200739

[bib140] Moya A , Ferrer M. Functional redundancy-induced stability of gut microbiota subjected to disturbance. Trends Microbiol. 2016;24:402–13.. 10.1016/j.tim.2016.02.002. 26996765

[bib142] Muller EEL , Faust K, Widder S et al. Using metabolic networks to resolve ecological properties of microbiomes. Curr Opin Syst Biol. 2018;8:73–80.. 10.1016/j.coisb.2017.12.004

[bib141] Muller EEL . Determining microbial niche breadth in the environment for better ecosystem fate predictions. mSystems. 2019;4:e00080–19.. 10.1128/mSystems.00080-19. 31186307 PMC6584867

[bib143] Naeem U , Qazi MA. Leading edges in bioremediation technologies for removal of petroleum hydrocarbons. Environ Sci Pollut Res. 2020;27:27370–82.. 10.1007/s11356-019-06124-8. 31392621

[bib144] Nguyen NH , Song Z, Bates ST et al. FUNGuild: an open annotation tool for parsing fungal community datasets by ecological guild. Fung Ecol. 2016;20:241–8.. 10.1016/j.funeco.2015.06.006

[bib145] Niarakis A , Helikar T. A practical guide to mechanistic systems modeling in biology using a logic-based approach. Brief Bioinf. 2021;22:4. 10.1093/bib/bbaa236. PMC8293813 33064138

[bib146] Nivetha N , Srivarshine B, Sowmya B et al. A comprehensive review on bio-stimulation and bio-enhancement towards remediation of heavy metals degeneration. Chemosphere. 2023;312:137099. 10.1016/j.chemosphere.2022.137099. 36372332

[bib147] Nordberg M , Templeton DM, Andersen O, Duffus JH. Glossary of terms used in ecotoxicology (IUPAC recommendations). Pure Appl Chem. 2009;81:2009–970.. 10.1351/PAC-REC-08-07-09.

[bib148] Noyer M , Bernard M, Verneau O et al. Insights on the particle-attached riverine archaeal community shifts linked to seasons and to multipollution during a Mediterranean extreme storm event. Environ Sci Pollut Res. 2023;30. 10.1007/s11356-023-25637-x. 36780079

[bib149] Noyer M , Reoyo-Prats B, Aubert D et al. Particle-attached riverine bacteriome shifts in a pollutant-resistant and pathogenic community during a Mediterranean extreme storm event. Sci Total Environ. 2020;732:art. 139047. 10.1016/j.scitotenv.2020.139047. 32473395

[bib150] Nwankwegu AS , Zhang L, Xie D et al. Bioaugmentation as a green technology for hydrocarbon pollution remediation. Problems and prospects. J Environ Manag. 2022;304:114313. 10.1016/j.jenvman.2021.114313. 34942548

[bib151] O’Brien AM , Lins TF, Yang Y et al. Microplastics shift impacts of climate change on a plant-microbe mutualism: temperature, CO(2), and tire wear particles. Environ Res. 2022;203:111727. 10.1016/j.envres.2021.111727. 34339696

[bib155] Ockleford C , Adriaanse P, Berny P et al. Scientific opinion addressing the state of the science on risk assessment of plant protection products for in-soil organisms. EFSA J. 2017;15:e04690. 10.2903/j.efsa.2017.4690. 32625401 PMC7009882

[bib152] OECD . Test no. 471: bacterial reverse mutation test, OECD guidelines for the testing of chemicals, section 4. Paris: Éditions OECD, 2020. 10.1787/9789264071247-en.

[bib153] OECD . Test no. 216: soil microorganisms: nitrogen transformation test, OECD guidelines for the testing of chemicals, section 2. Paris: Éditions OECD, 2000a. 10.1787/9789264070226-en.

[bib154] OECD . Test no. 217: soil microorganisms: carbon transformation test, OECD guidelines for the testing of chemicals, section 2. Paris: Éditions OECD, 2000a. 10.1787/9789264070240-en.

[bib156] Orland C , Emilson EJS, Basiliko N et al. Microbiome functioning depends on individual and interactive effects of the environment and community structure. ISME J. 2019;13:1–11.. 10.1038/s41396-018-0230-x. 30042502 PMC6298968

[bib157] Parales RE , Haddock JD. Biocatalytic degradation of pollutants. Curr Opin Biotechnol. 2004;15:374–9.. 10.1016/j.copbio.2004.06.003. 15296933

[bib158] Peixoto RS , Voolstra CR, Sweet M et al. Harnessing the microbiome to prevent global biodiversity loss. Nat Microbiol. 2022;7:1726–35.. 10.1038/s41564-022-01173-1. 35864220

[bib159] Persson L , Carney Almroth BM, Collins CD et al. Outside the safe operating space of the planetary boundary for novel entities. Environ Sci Technol. 2022;56, 3–21., 10.1021/acs.est.1c04158. 35038861 PMC8811958

[bib161] Pesce S , Ghiglione J-F, Topp E et al. Editorial: microbial ecotoxicology. Front Microbiol. 2020a;11:1342. 10.3389/fmicb.2020.01342. 32676059 PMC7333374

[bib160] Pesce S , Campiche S, Casado-Martinez C et al. Towards simple tools to assess functional effects of contaminants on natural microbial and invertebrate sediment communities. Environ Sci Pollut Res. 2020b;27:6680–9.. 10.1007/s11356-019-07331-z. 31863366

[bib220_1694761880716] Pesce S . Bérard A, Coutellec MA et al. Linking ecotoxicological effects on biodiversity and ecosystem functions to impairment of ecosystem services is a challenge: an illustration with the case of plant protection products. Environ Sci Pollut Res. 2023; 10.1007/s11356-023-29128-x 37548787

[bib162] Petric I , Karpouzas DG, Bru D et al. Nicosulfuron application in agricultural soils drives the selection towards NS-tolerant microorganisms harboring various levels of sensitivity to nicosulfuron. Environ Sci Pollut Res. 2016;23:4320–33.. 10.1007/s11356-015-5645-6. 26517995

[bib163] Pettorelli N , Schulte to Bühne H et al. Satellite remote sensing of ecosystem functions: opportunities, challenges and way forward. Remote Sens Ecol Conserv. 2018;4:71–93.. 10.1002/rse2.59.

[bib164] Picek L , Šulc M, Matas J et al., Automatic fungi recognition: deep learning meets mycology. Sensors. 2022;22:2. 10.3390/s22020633. PMC8779018 35062595

[bib165] Pinyayev TS , Kohan MJ, Herbin-Davis K et al. Preabsorptive metabolism of sodium arsenate by anaerobic microbiota of mouse cecum forms a variety of methylated and thiolated arsenicals. Chem Res Toxicol. 2011;24:475–7.. 10.1021/tx200040w. 21388151

[bib166] Põlme S , Abarenkov K, Henrik Nilsson R et al. FungalTraits: a user-friendly traits database of fungi and fungus-like stramenopiles. Fung Diver. 2020;105:1–16.. 10.1007/s13225-020-00466-2.

[bib167] Prygiel J , Coste M. Mise au point de l’Indice Biologique Diatomée, un indice diatomique pratique applicable au réseau hydrographique français. L’Eau, l’industrie, les nuisances. 1998;211:40–5.

[bib168] Rahman Z , Singh VP. Bioremediation of toxic heavy metals (THMs) contaminated sites: concepts, applications and challenges. Environ Sci Pollut Res. 2020;27:27563–581.. 10.1007/s11356-020-08903-0. 32418096

[bib169] Ranjan R , Rani A, Metwally A et al. Analysis of the microbiome: advantages of whole genome shotgun versus 16S amplicon sequencing. Biochem Biophys Res Commun. 2016;469:967–77.. 10.1016/j.bbrc.2015.12.083. 26718401 PMC4830092

[bib170] Romillac N , Santorufo L. Transferring concepts from plant to microbial ecology: a framework proposal to identify relevant bacterial functional traits. Soil Biol Biochem. 2021;162:108415. 10.1016/j.soilbio.2021.108415.

[bib171] Sabater S , Elosegi A, Ludwig R. Chapter 21 – summary, implications and recommendations for the occurrence and effects of multiple stressors in river ecosystems. In: Sabater S, Elosegi A, Ludwig R, (eds), Multiple Stressors in River Ecosystems. Amsterdam: Elsevier, 2019, 375–80.. 10.1016/B978-0-12-811713-2.00021-2.

[bib172] Sagan V , Peterson KT, Maimaitijiang M et al. Monitoring inland water quality using remote sensing: potential and limitations of spectral indices, bio-optical simulations, machine learning, and cloud computing. Earth Sci Rev. 2020;205:103187. 10.1016/j.earscirev.2020.103187.

[bib173] Santillan E , Seshan H, Constancias F et al. Frequency of disturbance alters diversity, function, and underlying assembly mechanisms of complex bacterial communities. npj Biofilms Microbiomes. 2019;5:8. 10.1038/s41522-019-0079-4. 30774969 PMC6370796

[bib174] Saravanan A , Kumar PS, Vo D-VN et al. A review on catalytic-enzyme degradation of toxic environmental pollutants: microbial enzymes. J Hazard Mater. 2021;419:126451. 10.1016/j.jhazmat.2021.126451. 34174628

[bib175] Seneviratne CJ , Suriyanarayanan T, Widyarman AS et al. Multi-omics tools for studying microbial biofilms: current perspectives and future directions. Crit Rev Microbiol. 2020;46:759–78.. 10.1080/1040841X.2020.1828817. 33030973

[bib176] Seymour M . Rapid progression and future of environmental DNA research. Commun Biol. 2019;2:80. 10.1038/s42003-019-0330-9. 30820475 PMC6393415

[bib177] Sharma B , Dangi AK, Shukla P. Contemporary enzyme based technologies for bioremediation: a review. . Environ Manage. 2018;210:10–22.. 10.1016/j.jenvman.2017.12.075. 29329004

[bib178] Sharma P . Efficiency of bacteria and bacterial assisted phytoremediation of heavy metals: an update. Bioresour Technol. 2021;328:124835. 10.1016/j.biortech.2021.124835. 33618184

[bib180] Shi Z , Yin H, et al., Van Nostrand Functional gene array-based ultrasensitive and quantitative detection of microbial populations in complex communities. mSystems. 2019;4:e00296–19.. 10.1128/mSystems.00296-19. 31213523 PMC6581690

[bib128] Shukla M . Microbial nanotechnology for bioremediation of industrial wastewater. Front Microbiol. 2020;11:590631. 10.1007/978-3-031-24086-7_14. 33224126 PMC7667373

[bib181] Simonin M , Richaume A, Guyonnet JP et al. Titanium dioxide nanoparticles strongly impact soil microbial function by affecting archaeal nitrifiers. Sci Rep. 2016;6:art. 33643. 10.1038/srep33643. PMC5034236 27659196

[bib182] Singh AK , Bilal M, Iqbal HMN et al. Trends in predictive biodegradation for sustainable mitigation of environmental pollutants: recent progress and future outlook. Sci Total Environ. 2021;770:144561. 10.1016/j.scitotenv.2020.144561. 33736422

[bib183] Sweeney CJ , Bottoms M, Ellis S et al. Arbuscular mycorrhizal fungi and the need for a meaningful regulatory plant protection product testing strategy. Environ Toxicol Chem. 2022;41:1808–23.. 10.1002/etc.5400. 35678214 PMC9543394

[bib184] Tahon G , Geesink P, Ettema TJG. Expanding archaeal diversity and phylogeny: past, present, and future. Annu Rev Microbiol. 2021;75:359–81.. 10.1146/annurev-micro-040921-050212. 34351791

[bib185] Tapolczai K , Keck F, et al., Bouchez Diatom DNA metabarcoding for biomonitoring: strategies to avoid major taxonomical and bioinformatical biases limiting molecular indices capacities. Front Ecol Evol. 2019;7:409. 10.3389/fevo.2019.00409.

[bib186] Tapolczai K , Selmeczy GB, Szabó B et al. The potential of exact sequence variants (ESVs) to interpret and assess the impact of agricultural pressure on stream diatom assemblages revealed by DNA metabarcoding. Ecol Indic. 2021;122:107322. 10.1016/j.ecolind.2020.107322.

[bib187] Tardy V , Mathieu O, Leveque J et al. Stability of soil microbial structure and activity depends on microbial diversity. Environ Microbiol Rep. 2014;6:173–83.. 10.1111/1758-2229.12126. 24596291

[bib188] Thiele-Bruhn S . The role of soils in provision of genetic, medicinal and biochemical resources. Philos Trans R Soc B Biol Sci. 2021;376:20200183. 10.1098/rstb.2020.0183. PMC8349636 34365823

[bib189] Thijs S , Sillen W, Rineau F et al. Towards an enhanced understanding of plant–microbiome interactions to improve phytoremediation: engineering the metaorganism. Front Microbiol. 2016;7:341. https://doi.org/.3389/fmicb.2016.00341. 27014254 10.3389/fmicb.2016.00341 PMC4792885

[bib190] Thiour-Mauprivez C , Martin-Laurent F, Calvayrac C, Barthelmebs L. Effects of herbicide on non-target microorganisms: towards a new class of biomarkers?. Sci Total Environ. 2019;684:314–25.. 10.1016/j.scitotenv.2019.05.230. 31153078

[bib191] Thomas F , Corre E, Cébron A. Stable isotope probing and metagenomics highlight the effect of plants on uncultured phenanthrene-degrading bacterial consortium in polluted soil. ISME J. 2019;13:1814–30.. 10.1038/s41396-019-0394-z. 30872807 PMC6775975

[bib192] Tlili A , Berard A, Blanck H et al. Pollution-induced community tolerance (PICT): towards an ecologically relevant risk assessment of chemicals in aquatic systems. Freshwat Biol. 2016;61:2141–51.. 10.1111/fwb.12558.

[bib193] Tlili A , Montuelle B, Triquet CA et al. Microbial Pollution-Induced Community Tolerance. Boca Raton: CRC Press, 2011. 10.1201/b10519-5.

[bib194] Trevelline BK , Fontaine SS, Hartup BK et al. Conservation biology needs a microbial renaissance: a call for the consideration of host-associated microbiota in wildlife management practices. Proc R Soc B Biol Sci. 2019;286:20182448. 10.1098/rspb.2018.2448. PMC6364583 30963956

[bib195] Uria N , Fiset E, Pellitero MA et al. Immobilisation of electrochemically active bacteria on screen-printed electrodes for rapid in situ toxicity biosensing. Environ Sci Ecotechnol. 2020;3:100053. 10.1016/j.ese.2020.100053. 36159604 PMC9488082

[bib196] Valdivia-Rivera S , Ayora-Talavera T, Lizardi-Jiménez MA et al. Encapsulation of microorganisms for bioremediation: techniques and carriers. Rev Environ Sci Bio/Technol. 2021;20:815–38.. 10.1007/s11157-021-09577-x.

[bib197] van der Plas F . Biodiversity and ecosystem functioning in naturally assembled communities. Biol. 2019;94:1220–45.. 10.1111/brv.12499. 30724447

[bib198] Veloso S , Amouroux D, Lanceleur L et al. Keystone microbial taxa organize micropollutant-related modules shaping the microbial community structure in estuarine sediments. J Hazard Mater. 2023;448:130858. 10.1016/j.jhazmat.2023.130858. 36706488

[bib199] Venkatachalapathy R , Karthikeyan P. Application of diatom-based indices for monitoring environmental quality of riverine ecosystems: a review. In: Ramkumar M, Kumaraswamy K, Mohanraj R, (eds), Environmental Management of River Basin Ecosystems. Cham: Springer International Publishing, 2015, 593–619.. 10.1007/978-3-319-13425-3_28.

[bib200] Venkataramanan A , Faure-Giovagnoli P, Regan C et al. Usefulness of synthetic datasets for diatom automatic detection using a deep-learning approach. Eng Appl Artif Intell. 2023;117:105594. 10.1016/j.engappai.2022.105594.

[bib201] Vijayaraj V , Laviale M, Allen J et al. Multiple-stressor exposure of aquatic food webs: nitrate and warming modulate the effect of pesticides. Water Res. 2022;216:118325. 10.1016/j.watres.2022.118325. 35349923

[bib202] Virta L , Teittinen A. Threshold effects of climate change on benthic diatom communities: evaluating impacts of salinity and wind disturbance on functional traits and benthic biomass. Sci Total Environ. 2022;826:154130. 10.1016/j.scitotenv.2022.154130. 35219662

[bib203] Virta L. , Soininen J., Norkko A. Diversity and distribution across a large environmental and spatial gradient: evaluating the taxonomic and functional turnover, transitions and environmental drivers of benthic diatom communities. Glob Ecol Biogeogr. 2020;29:2214–28.. 10.1111/geb.13190.

[bib204] Wagg C , Schlaeppi K, Banerjee S et al. Fungal-bacterial diversity and microbiome complexity predict ecosystem functioning. Nat Commun. 2019;10:4841. 10.1038/s41467-019-12798-y. 31649246 PMC6813331

[bib205] Wakelin S , Lombi E, Donner E et al. Application of MicroResp™ for soil ecotoxicology. Environ Pollut. 2013;179:177–84.. 10.1016/j.envpol.2013.04.010. 23685630

[bib206] Walker JR , WoodsAC, PierceMKet al. Functionally diverse microbial communities show resilience in response to a record-breaking rain event. ISME Commun. 2022;2:81. 10.1038/s43705-022-00162-z.37938674

[bib207] Wang J , Guo X, Xue J. Biofilm-developed microplastics as vectors of pollutants in aquatic environments. Environ Sci Technol. 2021;55:12780–90.. 10.1021/acs.est.1c04466. 34553907

[bib208] Westoby M , Gillings MR, Madin JS et al. Trait dimensions in bacteria and archaea compared to vascular plants. Ecol Lett. 2021;24:1487–504.. 10.1111/ele.13742. 33896087

[bib209] Xiao E , Ning Z, Xiao T et al. Soil bacterial community functions and distribution after mining disturbance. Soil Biol Biochem. 2021;157:108232. 10.1016/j.soilbio.2021.108232.

[bib210] Xie Y , Dong H, Zeng G et al. The interactions between nanoscale zero-valent iron and microbes in the subsurface environment: a review. J Hazard Mater. 2017;321:390–407.. 10.1016/j.jhazmat.2016.09.028. 27669380

[bib211] Xu L , Xu L, Chen Y et al. Accurate classification of algae using deep convolutional neural network with a small database. ACS ES&T Water. 2022;2:1921–8.. 10.1021/acsestwater.1c00466.

[bib212] Yang L , Wang J, Yang Y et al. Phytoremediation of heavy metal pollution: hotspots and future prospects. Ecotoxicol Environ Saf. 2022;234:113403. 10.1016/j.ecoenv.2022.113403. 35286961

[bib213] Yergeau E , Arbour M, Brousseau R et al. Microarray and real-time PCR analyses of the responses of High-Arctic soil bacteria to hydrocarbon pollution and bioremediation treatments. Appl Environ Microbiol. 2009;75:6258–67.. 10.1128/AEM.01029-09. 19684169 PMC2753079

[bib214] Yu KSH , Wong AHY, Yau KWY et al. Natural attenuation, biostimulation and bioaugmentation on biodegradation of polycyclic aromatic hydrocarbons (PAHs) in mangrove sediments. Mar Pollut Bull. 2005;51:1071–7.. 10.1016/j.marpolbul.2005.06.006. 16023146 PMC2885898

[bib215] Yuan J , Shentu J, Ma B et al. Microbial and abiotic factors of flooded soil that affect redox biodegradation of lindane. Sci Total Environ. 2021;780:146606. 10.1016/j.scitotenv.2021.146606. 34030285

[bib216] Zandalinas SI , Fritschi FB, Mittler R. Global warming, climate change, and environmental pollution: recipe for a multifactorial stress combination disaster. Trends Plant Sci. 2021;26:588–99.. 10.1016/j.tplants.2021.02.011. 33745784

[bib217] Zhang F , Battaglia-Brunet F, Hellal J et al. Impact of Fe(III) (oxyhydr)oxides mineralogy on iron solubilization and associated microbial communities. Front Microbiol. 2020;11:571244. 10.3389/fmicb.2020.571244. 33329429 PMC7715016

[bib218] Zhang S , Hu Z, Wang H. Metagenomic analysis exhibited the co-metabolism of polycyclic aromatic hydrocarbons by bacterial community from estuarine sediment. Environ Int. 2019;129:308–19.. 10.1016/j.envint.2019.05.028. 31150973

[bib219] Zhou T , Han H, Liu P et al. Microbial fuels cell-based biosensor for toxicity detection: a review. Sensors. 2017;17. 10.3390/s17102230. PMC5677232 28956857

[bib220] Zhou Y , Kumar M, Sarsaiya S et al. Challenges and opportunities in bioremediation of micro-nano plastics: a review. Sci Total Environ. 2022;802:149823. 10.1016/j.scitotenv.2021.149823. 34454140

