# Correction to: Unlocking secrets of microbial ecotoxicology: recent achievements and future challenges

**DOI:** 10.1093/femsec/fiad135

**Published:** 2023-11-06

This is a correction to: Jennifer Hellal, Lise Barthelmebs, Annette Bérard, Aurélie Cébron, Giulia Cheloni, Simon Colas, Cristiana Cravo-Laureau, Caroline De Clerck, Nicolas Gallois, Marina Hery, Fabrice Martin-Laurent, Jean Martins, Soizic Morin, Carmen Palacios, Stéphane Pesce, Agnès Richaume, Stéphane Vuilleumier, Unlocking secrets of microbial ecotoxicology: recent achievements and future challenges, *FEMS Microbiology Ecology*, Volume 99, Issue 10, October 2023, fiad102, https://doi.org/10.1093/femsec/fiad102

In the originally published version of this manuscript, the names of all co-authors except for the first author were erroneously transposed. These names should read: “Lise Barthelmebs, Annette Bérard, Aurélie Cébron, Giulia Cheloni, Simon Colas, Cristiana Cravo-Laureau, Caroline De Clerck, Nicolas Gallois, Marina Hery, Fabrice Martin-Laurent, Jean Martins, Soizic Morin, Carmen Palacios, Stéphane Pesce, Agnès Richaume, Stéphane Vuilleumier” instead of: “Barthelmebs Lise, Bérard Annette, Cébron Aurélie, Cheloni Giulia, Colas Simon, Cravo-Laureau Cristiana, De Clerck Caroline, Gallois Nicolas, Hery Marina, Martin-Laurent Fabrice, Martins Jean, Morin Soizic, Palacios Carmen, Pesce Stéphane, Richaume Agnès, Vuilleumier Stéphane”.

This error has been corrected in the article.

